# Dynamic changes in genomic 5-hydroxymethyluracil and N6-methyladenine levels in the *Drosophila melanogaster* life cycle and in response to different temperature conditions

**DOI:** 10.1038/s41598-022-22490-9

**Published:** 2022-10-20

**Authors:** Marta Starczak, Maciej Gawronski, Aleksandra Wasilow, Pawel Mijewski, Ryszard Olinski, Daniel Gackowski

**Affiliations:** Department of Clinical Biochemistry, Faculty of Pharmacy, Collegium Medicum in Bydgoszcz, Nicolaus Copernicus University in Toruń, 85-092 Bydgoszcz, Poland

**Keywords:** DNA, Epigenetics

## Abstract

In this study, the level of DNA modifications was investigated in three developmental stages of *Drosophila melanogaster* (larvae, pupae, imago) and in an in vitro model (Schneider 2 cells). Analysis was carried out using two-dimensional ultra-performance liquid chromatography with tandem mass spectrometry. Our method made it possible, for the first time, to analyze a broad spectrum of DNA modifications in the three stages of *Drosophila*. Each stage was characterized by a specific modification pattern, and the levels of these compounds fluctuated throughout the *D. melanogaster* life cycle. The level of DNA modification was also compared between insects bred at 25 °C (optimal temperature) and at 18 °C, and the groups differed significantly. The profound changes in N6-methyladenine and 5-hydroxymethyluracil levels during the *Drosophila* life cycle and as a result of breeding temperature changes indicate that these DNA modifications can play important regulatory roles in response to environmental changes and/or biological conditions. Moreover, the supplementation of Schneider 2 cells with 1 mM L-ascorbic acid caused a time-dependent increase in the level of 5-(hydroxymethyl)-2′-deoxyuridine. These data suggest that a certain pool of this compound may arise from the enzymatic activity of the dTET protein.

## Introduction

The fruit fly (*Drosophila melanogaster*) is a well-known model organism used in a vast spectrum of research fields ranging from fundamental genetics to the development of tissues and organs. This species is also used to reveal molecular mechanisms that have direct implications for human immunity and health, including cancer research^1,2^. Flies are relatively easy to rear in the laboratory; their life cycle is short (it takes approximately 9–10 days to develop from a fertilized egg to an adult at 25 °C), and they have many offspring, ensuring that a large amount of material necessary for research is obtained in a short time^3^. Importantly, the *Drosophila* genome is 60% homologous to the human genome, and approximately 75% of the genes associated with human disease have homologs in the fruit fly^2,4^.

Epigenetic DNA modifications, such as DNA methylation, can expand a genome’s regulatory flexibility, which may contribute to the evolution of phenotypic plasticity, an important biological phenomenon that allows organisms with the same genotype to respond adaptively to the variable environment^5^. Methyl groups can be added directly to cytosines in CpG dinucleotides, but methylation can occur on other cytosines or even other nucleotides. The enzymatic addition of methyl groups to nucleotides involves several DNA methyltransferases (DNMTs). Mammals have a complete set of methyltransferases: DNMT1, DNMT2 and DNMT3 (A and B). DNMT1 is responsible for maintaining the methylation pattern during DNA replication. In contrast, DNMT3A and DNMT3B are de novo methyltransferases^6^. DNMT2 is not considered a true DNA methyltransferase and may be involved in the methylation of tRNAs^6,7^.

Only four insect orders were reported to possess a full set of methyltransferases (including the *Hymenoptera* to which *Apis mellifera* belongs). *D. melanogaster* fails to possess DNMT1 or DNMT3 orthologs. The genome of the fruit fly along with the whole order of *Diptera* contains only a single recognized DNMT ortholog of DMNT2 (dDNMT2)^6^. For this reason, there was a hypothesis for a long time that *D. melanogaster* did not contain 5-methylcytosine (5-mCyt) in its DNA. However, novel, more sensitive methods enabled to determine the low levels of 5-mCyt in *Drosophila*. The immunoprecipitation of methylated cytosines coupled with bisulfite sequencing shows that ~ 1% of the fly genome at embryo stage 5 is methylated at low levels^8^. Using methods based on liquid chromatography, Capuano et al. and Rasmussen et al. quantified 5-mCyt in adult flies^9,10^. The level of this modification ranged from 0.002% of all cytosines methylated in the female Oregon-R lab strain (liquid chromatography-tandem mass spectrometry) to 0.034% in the mixed population of male and female wt/w118 (liquid chromatography selective reaction monitoring)^9,10^. In 2018, Dashmukh et al. presented an analysis of the 5-mCyt level in all developmental stages of *D. melanogaster*. The highest level was observed in embryos, slightly lower in larvae; after metamorphosis, the content of this modification decreased significantly^11^. Interestingly, lines deficient for dDNMT2 in *Drosophila* retain genomic methylation, which implies the presence of a novel, cryptic DNA methyltransferase^8^. These results were confirmed by Panikar et al.^12^. These authors showed de novo DNA methyltransferase activity in adult *D. melanogaster* and the presence of CpC methylation in selected genomic regions^12^. Deshmukh et al. observed the presence of methylation in flies with a dDNMT2 knockout, although the methylation profile was altered^13^.

Methylation is also observed in the RNA of many species. The finding that dDNMT2 can methylate tRNAs led to the speculation that its primary function in *Drosophila* is RNA methylation^8,14,15^. As a result, it is possible that the 5-mCyt observed in *D. melanogaster* can be derived only from RNA. This theory was denied by Capuano et al.^9^. They described a method that enabled the separation of DNA-derived and RNA-derived 5-mCyt. These authors used a protocol involving the enzymatic hydrolysis of the genetic material to deoxynucleotides and quantified only 5-mCyt derived from the DNA. The authors confirmed that 5-mCyt was a rare DNA modification in *Drosophila* but absent in laboratory and industrial yeast species (*S. cerevisiae, S. pombe, S. boulardii, S. paradoxus*, and *P. pastoris*)^9^.

In 2015, the presence of another methylated DNA modification, namely, N6-methyladenine (N6-mAde), was found in *Drosophila* embryos and in the tissues of adult flies. The level of N6-mAde changed dynamically during embryonic development. The highest level was observed at the ∼ 0.75 h stage, and it decreased significantly at the 4–16 h stages. Additionally, low levels of N6-mAde were observed in tissues from adult flies^16^. N6-mAde was found to be enriched at intragenic regions, within introns and at simple repeats^15,17^.

Moreover, small amounts of 5-hydroxymethylcytosine (5-hmCyt) were also found in fruit fly DNA^10,16^. In mammals, 5-hmCyt is considered to be an important epigenetic modification and product of 5-mCyt oxidation by ten-eleven translocation (TET) enzymes. This modification may be further oxidized to 5-formylcytosine (5-fCyt) and 5-carboxylcytosine (5-caCyt). Then, the BER (base excision repair) pathway is induced by the involvement of thymine DNA glycosylase (TDG) to replace the above-described base modifications with cytosine (reviewed in^18^). Some evidence from experimental studies suggests that mammalian TET may also be involved in the synthesis of 5-hydroxymethyluracil (5-hmUra), a compound with potential epigenetic functions^19^. *D. melanogaster* also has a single ortholog of the aforementioned proteins (dTet). The expression of dTet is dynamic and strictly regulated during development. The highest levels were found in the central nervous system and the brain. However, it is also required in other tissues at specific developmental time points^15^. dTet is most similar to human TET3 and can oxidase 5-mCyt to 5-hmCyt^15,20^. Nevertheless, Zhang et al. provided evidence that dTet (called *Drosophila* DNA N6-mAde demethylase -DMAD) was an N6-mAde demethylase. DMAD catalyzed the demethylation of N6-mAde both in vitro and in vivo^15,16^.

Interestingly, dTet enzymes also catalyze the formation of 5-hmCyt in RNA. Hydroxymethylated RNA was found in *Drosophila* Schneider 2 (S2) cells and during the early embryogenesis of *D. melanogaster.* 5-HmCyt in RNA is most abundant in the *Drosophila* brain, and this modification may play a significant role in the proper development of the fruit fly brain^21,22^.

Muha et al. found that throughout the *D. melanogaster* larval stages, there was a drastic decline in deoxyuridine triphosphatase that occurred in parallel with the absence of uracil DNA N-glycosylase. Therefore, larval tissues may accumulate uracil in their DNA (paired with adenine). The relatively high levels of uracil-containing DNA are well tolerated in larval stages but corrected during the development process^23,24^. In fact, the DNA containing uracil was recognized and processed by the uracilated DNA degrading factor. This protein is undetectable in the embryonic stage, most larval stages and the imago but is strongly upregulated just before pupation^24–26^.

In this study, we analyzed the level of DNA modifications in an in vitro model (*Drosophila* Schneider 2 cell line) and at three developmental stages of the fruit fly (larvae, pupae, imago). The analysis was carried out on the whole-genome level using isotope-dilution automated online two-dimensional ultra-performance liquid chromatography with tandem mass spectrometry (2D-UPLC‒MS/MS) and stable isotope-labeled internal standards.

## Results

### Dynamics of DNA modification levels during the *Drosophila melanogaster* life cycle

In the DNA of *D. melanogaster* larvae, pupae and the mixed population of male and female imago, it was possible to quantify the levels of 5-methyl-2′-deoxycytidine (5-mdC), 5-(hydroxymethyl)-2′-deoxycytidine (5-hmdC), 5-(hydroxymethyl)-2′-deoxyuridine (5-hmdU), 2′-deoxyuridine (dU), 8-oxo-7,8-dihydro-2'-deoxyguanosine (8-oxodG) and N6-methyl-2′-deoxyadenosine (N6-mdA) (Supplementary Tables S1, S2). Despite the very high sensitivity of the applied method, two other potential dTet products (5-formyl-2′-deoxycytidine and 5-carboxy-2′-deoxycytidine) were not identified in the *Drosophila* genome.

**Figure 1:**
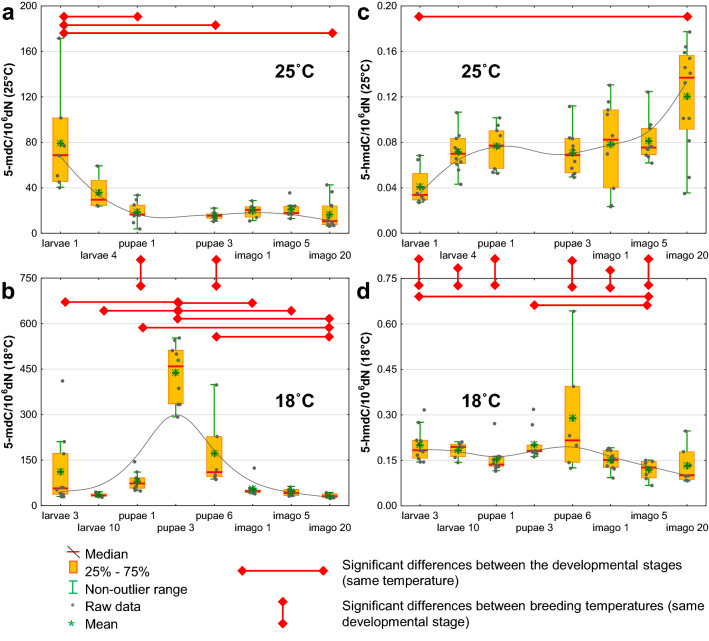
Changes in the levels of 5-methyl-2′-deoxycytidine (5-mdC) and 5-(hydroxymethyl)-2′-deoxycytidine (5-hmdC) during the *D. melanogaster* life cycle, (**a**) 5-methyl-2′-deoxycytidine in insects bred in the optimal temperature conditions (25 °C), (**b**) 5-methyl-2′-deoxycytidine in insects bred at 18 °C, (**c**) 5-(hydroxymethyl)-2′-deoxycytidine in insects bred in optimal temperature conditions (25 °C), (**d**) 5-(hydroxymethyl)-2′-deoxycytidine in insects bred at 18 °C. The results are presented as the mean and median values, interquartile ranges, and nonoutlier ranges (*p* < 0.05).

Interestingly, each developmental stage was characterized by a specific DNA modification pattern, and the level of these compounds fluctuated throughout the *D. melanogaster* life cycle. The level of DNA modification was also compared between insects bred under optimal temperature conditions (25 °C) and at 18 °C. The lower bred temperature resulted in a significant almost twofold extension of the cycle from laying eggs to the eclosion of first flies. Both groups differed significantly in the content of the described DNA modifications (Supplementary Table S3). In insects bred in optimal temperature conditions, the highest level of 5-mdC was observed in larvae, and subsequent development stages were characterized by decreasing levels of this modification (Fig. 1a and Supplementary Table S4). The opposite trend was observed for 5-hmdC levels (Fig. 1c and Supplementary Table S4). Interestingly, in individuals bred at 18 °C, a different pattern of these modifications was found. The highest levels of 5-mdC and 5-hmdC were observed in pupae, and the lowest levels were observed in 20-day-old flies (Fig. 1b,d and Supplementary Table S4).

**Figure 2:**
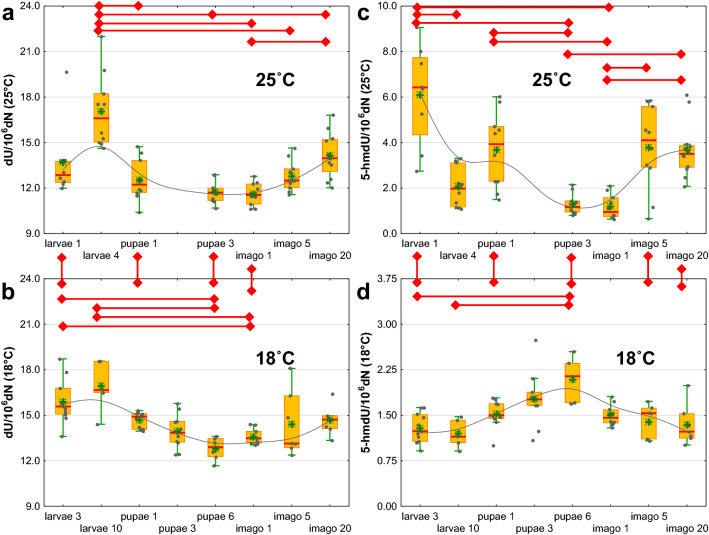
Changes in the levels of 2′-deoxyuridine (dU) and 5-(hydroxymethyl)-2′-deoxyuridine (5-hmdU) during the *D. melanogaster* life cycle (**a**) 2′-deoxyuridine in insects bred in the optimal temperature conditions (25 °C), (**b**) 2′-deoxyuridine in insects bred at 18 °C, (**c**) 5-(hydroxymethyl)-2′-deoxyuridine in insects bred in optimal temperature conditions (25 °C), (**d**) 5-(hydroxymethyl)-2′-deoxyuridine in insects bred at 18 °C. The results are presented as the mean and median values, interquartile ranges, and nonoutlier ranges (*p* < 0.05).

Regardless of the bred temperature, a similar pattern of fluctuations in the dU content was observed. The highest level of this modification was found in larvae (larvae 4 and larvae 10), after which the level decreased in pupae to grow again in adult flies (Fig. 2a,b and Supplementary Table S5). Interestingly, 1-day-old flies bred at 25 °C were characterized by a significantly lower level of dU than flies 20 days after eclosion (Fig. 2a and Supplementary Table S5). Under optimal conditions, the highest level of 5-hmdU was observed in larvae 1–2 days after hatching, while the lowest was observed in pupae just before turning into an adult insect (pupae 3) and in 1-day-old imago. It is worth noting that there were significant differences in the content of 5-hmdU between larvae of different ages (larvae 1 and larvae 4) and pupae of different ages (pupae 1 and pupae 3), as well as between 1-day-old imagos and flies 5 and 20 days after eclosion (Fig. 2c and Supplementary Table S5). A different pattern of changes in the level of 5-hmdU was observed in insects bred at 18 °C. The larvae had the lowest content of this modification, and the pupae just before turning into imago had the highest (Fig. 2d and Supplementary Table S5).

**Figure 3:**
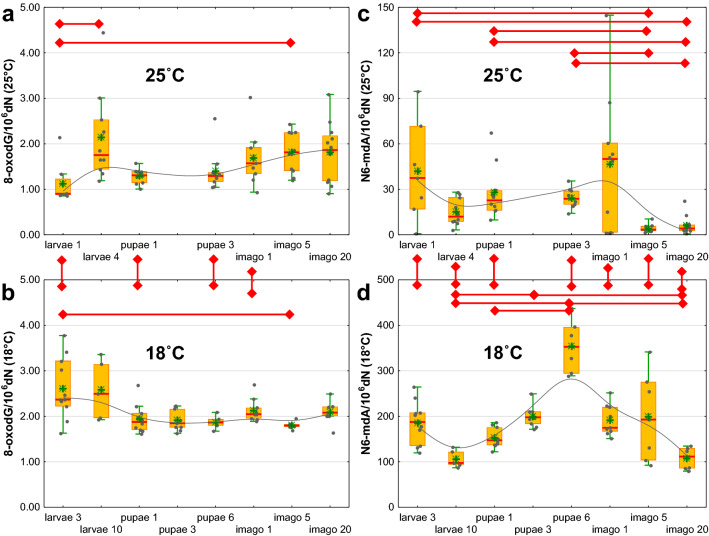
Changes in the levels of 8-oxo-7,8-dihydro-2′-deoxyguanosine (8-oxodG) and N6-methyl-2′-deoxyadenosine (N6-mdA) during the *D. melanogaster* life cycle (**a**) 8-oxo-7,8-dihydro-2′-deoxyguanosine in insects bred in optimal temperature conditions (25 °C), (**b**) 8-oxo-7,8-dihydro-2′-deoxyguanosine in insects bred at 18 °C, (**c**) N6-methyl-2′-deoxyadenosine in insects bred in optimal temperature conditions (25 °C), (**d**) N6-methyl-2′-deoxyadenosine in insects bred at 18 °C. The results are presented as the mean and median values, interquartile ranges, and nonoutlier ranges (*p* < 0.05).

The larvae 1–2 days after hatching (25 °C) had the lowest level of 8-oxodG, while the highest level of this modification was observed in the larvae 4 group. A relatively high 8-oxodG content was also found in adult *D. melanogaster* (imago 5 and imago 20) (Fig. 3a and Supplementary Table S6). In insects bred at 18 °C, a higher level of 8-oxodG was observed in the larvae, while the remaining development stages were characterized by a relatively constant content of this modification (Fig. 3b and Supplementary Table S6).

The highest levels of N6-mdA in insects bred at the optimal temperature were observed in larvae 1–2 days after hatching and in imagos 1 day after eclosion. Interestingly, the lowest level of this modification was found in 5-day-old and 20-day-old flies (Fig. 3c and Supplementary Table S6). The pupae 6 group bred at 18 °C had the highest level of N6-mdA. The lowest contents of this modification were observed in 10-day-old larvae and 20-day-old flies. There was also a significant difference in the level of N6-mdA between pupae 1 and 6 (Fig. 3d and Supplementary Table S6).

Additionally, the L-ascorbic acid (L-AA) content was measured in three developmental stages of *D. melanogaster*: larvae, pupae, and imagos. The highest levels were observed in pupae and the lowest in larvae (Supplementary Fig. S1).

### Levels of DNA modifications in in vitro model experiments

**Figure 4:**
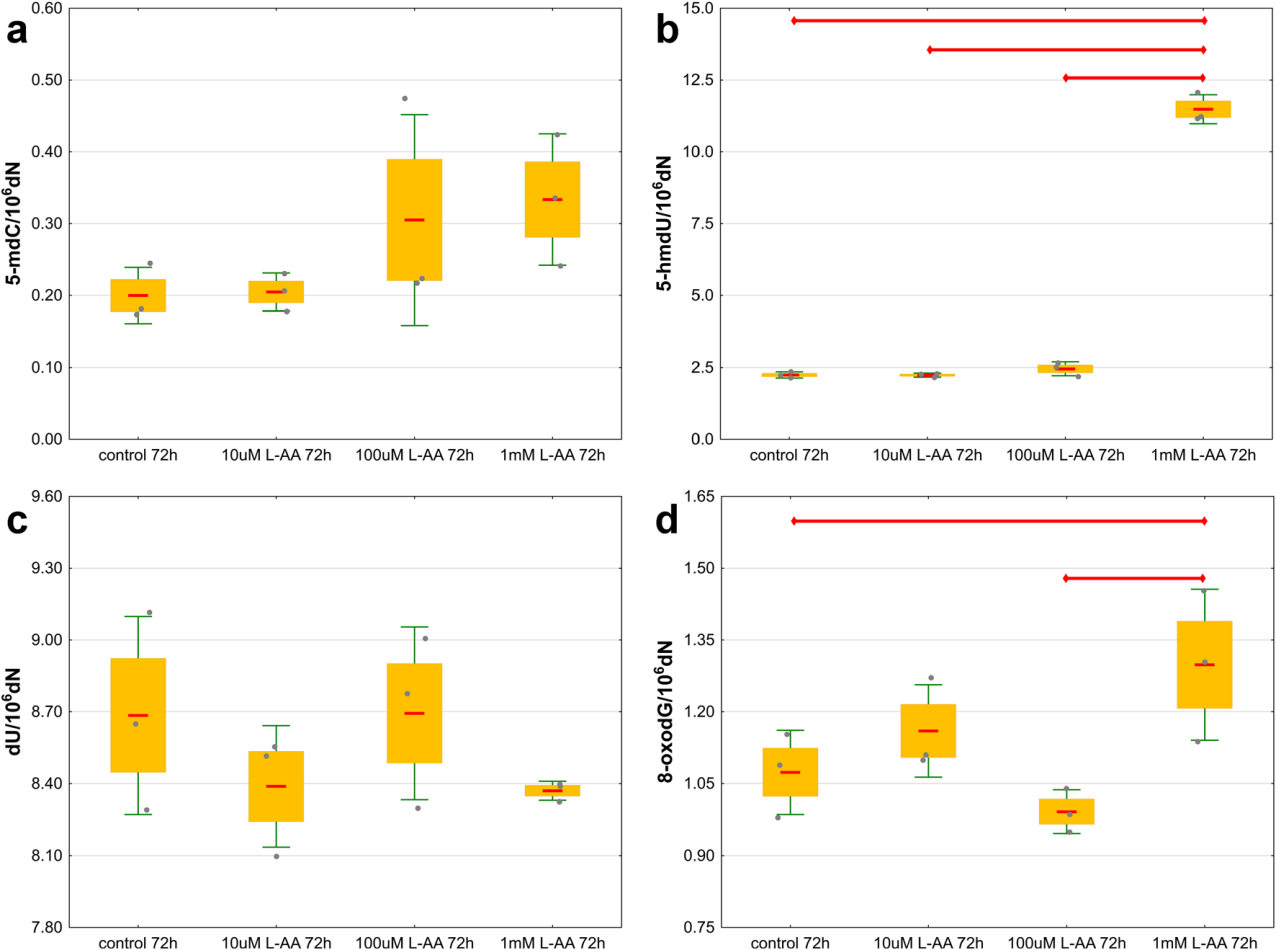
The levels of DNA modifications in S2 cells cultured in medium supplemented with different concentrations of L-ascorbic acid (**a**) 5-methyl-2′-deoxycytidine (5-mdC), (**b**) 5-(hydroxymethyl)-2′-deoxyuridine (5-hmdU), (**c**) 2′-deoxyuridine (dU), (**d**) 8-oxo-7,8-dihydro-2′-deoxyguanosine (8-oxodG). The results are presented as the mean, mean ± standard error and mean ± standard deviation (*p* < 0.05).

A number of experiments were carried out using an in vitro model. *Drosophila* S2 cells were cultured with the addition of the known activator of TET proteins L-AA. In S2 cells, the levels of 5-mdC, 5-hmdU, dU and 8-oxodG were quantified. To determine the optimal concentration of L-AA, the culture medium was supplemented to final concentrations of 10 µM, 100 µM and 1 mM. Significantly higher levels of 5-hmdU and 8-oxodG were observed in the DNA of cells grown in medium supplemented with 1 mM L-AA compared to control cells. There were no differences in the levels of 5-mdC and dU (Fig. 4 and Supplementary Tables S7, S8).

**Figure 5:**
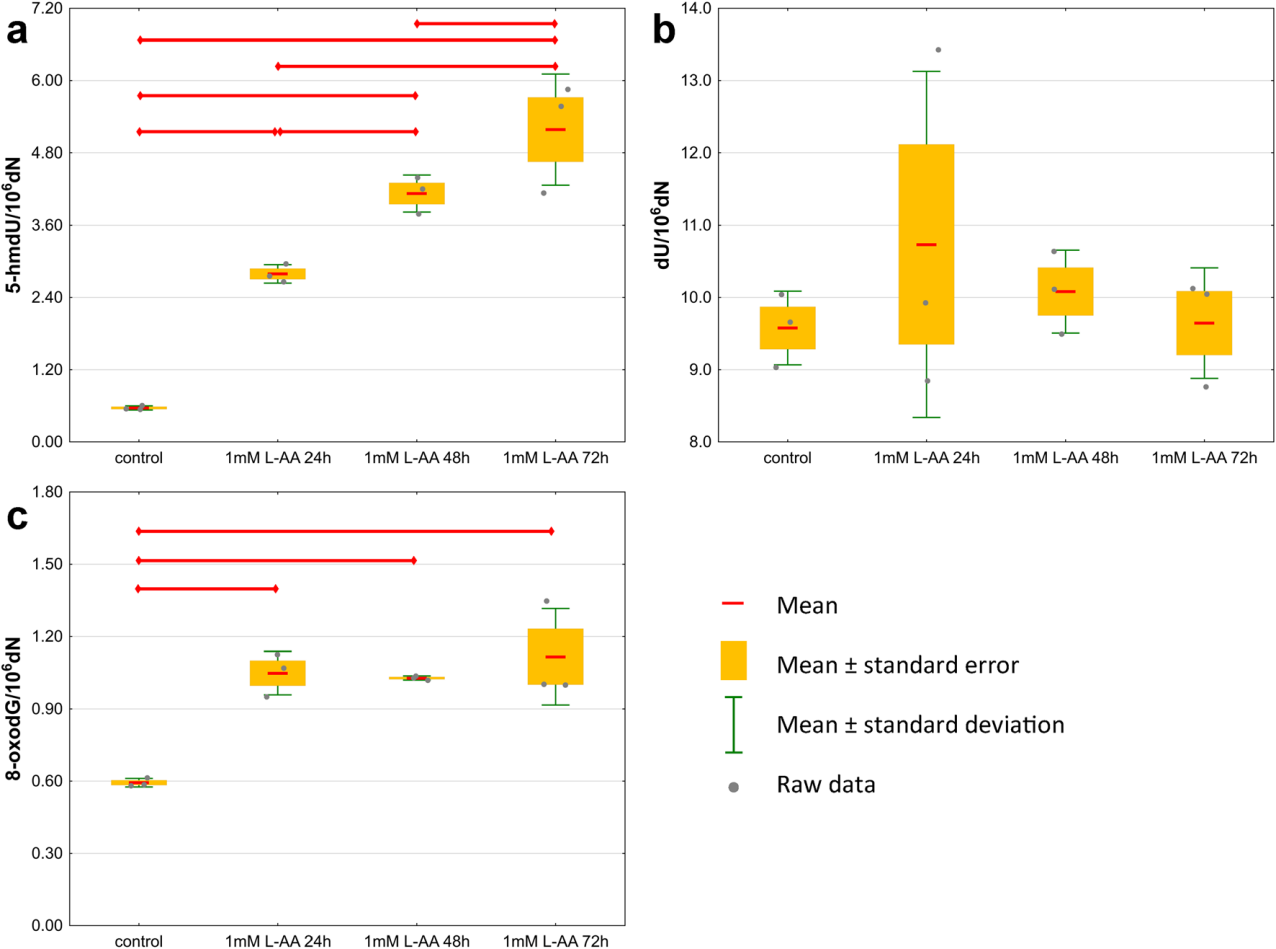
The levels of the DNA modifications in S2 cells cultured with the addition of 1 mM L-AA for 24 h, 48 h and 72 h (**a**) 5-(hydroxymethyl)-2′-deoxyuridine (5-hmdU), (**b**) 2′-deoxyuridine (dU), (**c**) 8-oxo-7,8-dihydro-2′-deoxyguanosine (8-oxodG). The results are presented as the mean, mean ± standard error and mean ± standard deviation (*p* < 0.05).

To evaluate the dynamics of the changes in the DNA modification content over time, S2 cells were cultured with the addition of 1 mM L-AA for 24 h, 48 h and 72 h. A time-dependent increase in the level of 5-hmdU and fluctuations in the 8-oxodG level were observed in supplemented cells compared to control cells (Fig. 5 and Supplementary Tables S9, S10).

**Figure 6:**
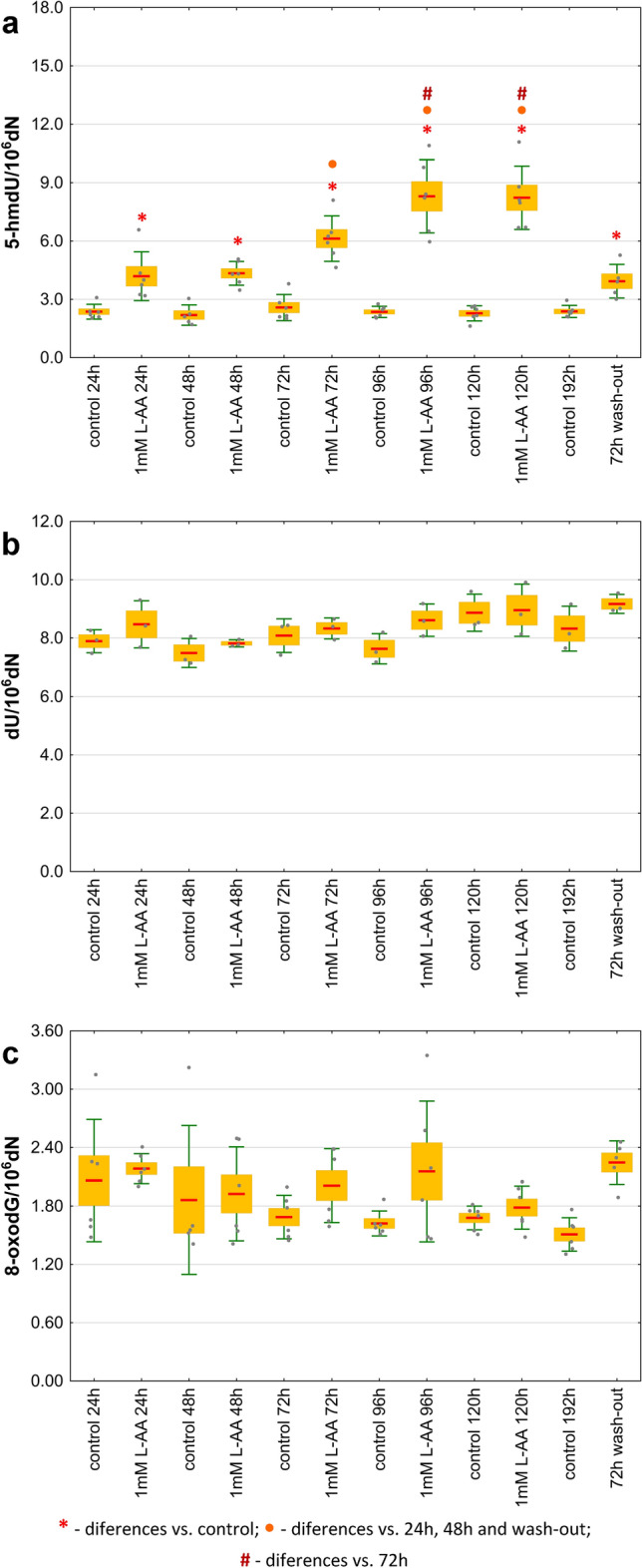
The kinetics of the DNA modification in S2 cells supplemented with 1 mM L-ascorbic acid and in control cells (**a**) 5-(hydroxymethyl)-2′-deoxyuridine (5-hmdU), (**b**) 2′-deoxyuridine (dU), (**c**) 8-oxo-7,8-dihydro-2′-deoxyguanosine (8-oxodG). The results are presented as the mean, mean ± standard error and mean ± standard deviation (*p* < 0.05).

**Figure 7:**
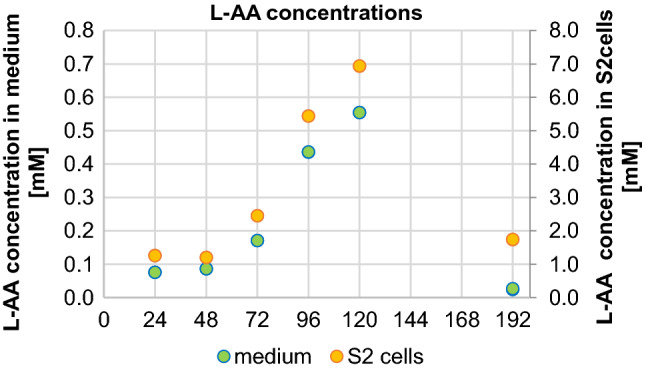
L-ascorbic acid concentration in supplemented *Drosophila* medium and in S2 cells.

In the subsequent experiment, the cultivation of S2 cells in medium supplemented with 1 mM L-AA was extended to 120 h (samples collected every 24 h), and after changing the medium (to nonsupplemented), the cells were cultured for another 72 h (72 h wash-out). The supplemented cells were characterized by a significantly higher level of 5-hmdU compared to the corresponding control cells. The same trend was also observed in cells after wash-out. The highest level of 5-hmdU was found after 96 h of culture in the supplemented medium, and it remained at a similar level after 120 h. After 72 h of wash-out, the content of this modification decreased to values slightly lower than those observed after 24 h of culture with the addition of 1 mM L-AA (Fig. 6 and Supplementary Tables S11, S12, S13, S14). Moreover, the level of L-AA was measured in the culture medium and in S2 cells. It was possible to determine changes in the ascorbate concentration over time in the treated cells and the medium. The concentration of ascorbic acid in control cells was below the detection limit of the method (Fig. 7).

## Discussion

Although *Drosophila* is a well-known and widely used model organism, relatively little is known about the processes of DNA methylation and demethylation in these insects. For many years, it was believed that 5-mCyt was not present in the fruit fly genome. The use of very sensitive methods made it possible to confirm the presence of this modification in the DNA of *Drosophila*. Several other DNA modifications (5-hmCyt, N6-mAde) were also found in the fruit fly. In this study, the changes in the content of DNA modifications during the *D. melanogaster* life cycle were analyzed. The isolation of genetic material from the various developmental stages of *Drosophila* is problematic. Both the commercially available kits tested by us and the protocols for manual extraction of nucleic acids previously developed for mammalian tissues failed in the case of the insect-derived DNA. For this reason, a new method of DNA isolation from *D. melanogaster* larvae, pupae and adult flies was developed. This method is based on a modified phenolic method, followed by the enzymatic hydrolysis of the acquired material to deoxynucleosides. Our DNA extraction method enabled us to obtain pure and highly polymerized DNA and, in turn, made it possible for the first time to analyze a broad spectrum of DNA modifications with high accuracy and precision in three developmental stages of the fruit fly.

Interestingly, each developmental stage was characterized by a specific DNA modification pattern, and the level of these compounds fluctuated throughout the *D. melanogaster* life cycle. Only the differences in the level of 5-mCyt in all the developmental stages of the fruit fly have been hitherto described^11^. The highest level of 5-mCyt was observed in embryos and larvae; after metamorphosis, the content of 5-mCyt decreased significantly (to ~ 0.001% 5-mCyt/Cyt in adult flies). Similar values were observed by Rasmussen et al. in the adult female Oregon-R lab strain^10^. In accordance with the reports of Deshmukh et al., the highest level of 5-mCyt in the DNA of 1-day-old larvae was found in this work, while in the subsequent developmental stages, the content of this modification was lower. The observed 5-mCyt levels are higher than those reported by Deshmukh et al. and Rasmussen et al. (in imago: ~ 0.01% 5-mCyt/Cyt versus 0.001–0.002% 5-mCyt/Cyt, respectively)^10,11^. However, Capuano et al. observed even higher methylation in adult flies (0.036% of all cytosines methylated)^9^. Curiously, we observed the opposite tendency (increase in the level in the subsequent development stages) in the case of the product of 5-mCyt demethylation, namely, 5-hmCyt. It is likely that *Drosophila* dTet is responsible for these changes. This protein can catalyze the oxidation of 5-mCyt to 5-hmCyt in the fruit fly^15,20^. Rasmussen et al. detected a small amount (0.15/10^6^ unmodified nucleosides) of 5-hmCyt in the DNA of adult flies^10^. We observed a similar level in the flies 20 days after eclosion. Additionally, Zhang et al. observed 5-hmCyt in tissues of adult flies, but at a much lower level (less than 20 modifications per genome)^16^.

We observed the highest level of 5-hmUra in the larvae (1–2 days after hatching) grown at 25 °C. The lowest level of this modification was observed in pupae just before pupation and in 1-day-old flies. Perhaps 5-hmUra plays a similar role to Ura during *Drosophila* development. 5-HmUra may be generated during the course of several processes (as reviewed in^27,28^). It was originally considered to be a product of a free radical reaction with thymine. Another potential source of this modification is the deamination of 5-hmCyt by activation-induced cytidine deaminase (AID/APOBEC). The lack of correlation between the level of 8-oxodG, a well-known marker of oxidative stress, and 5-hmUra (Supplementary Fig. S2), suggests that these modifications arise in different ways in *Drosophila*. Thus, the oxidation of thymine by free radicals as the main source of 5-hmUra can be excluded. Moreover, the very low level of 5-hmCyt (undetectable in S2 cells) in *D. melanogaster* suggests that deamination is also not responsible for the formation of 5-hmUra. Pfaffeneder et al. showed that 5-hmUra may be formed by the oxidation of thymine by TET enzymes^19^. Moreover, Pais et al. found that TET-like 5-mCyt oxygenase (NgTET1) in *Naegleria gruberi* is active against both 5-mCyt and thymine. Although the activity of NgTET1 toward thymine is much lower than that toward 5-mCyt, the authors confirmed the formation of 5-hmUra, 5-formyluracil and 5-carboxyuracil *in vitro*. Moreover, they showed that NgTET1 displays higher thymine-oxidation activity in vitro than mammalian TET1^29^.

Key factors in the mammalian demethylation process are 5-fCyt and 5-caCyt, which are removed from the DNA and replaced with unmodified cytosine. However, no such modifications in *D. melanogaster* DNA could be found. It is suggested that in *Drosophila*, 5-hmUra plays a significant role in the active demethylation process. It is possible that 5-hmUra initiates the processive demethylation of DNA, as proposed by Franchini et al.^30,31^. In line with this hypothesis, there is an alternative pathway, so-called processive DNA demethylation, which exists aside from the active process involved in local and specific DNA demethylation. According to the authors of this hypothesis, a single initiating event (such as a certain mismatch) may trigger the processive demethylation of numerous 5-mCyts (and perhaps also 5-hmCyts) on the same locus via long-path BER. Recent experiments with cell-free extracts and the circular heteroduplex DNA substrate demonstrated that 5-hmUra may trigger the removal of distant epigenetic modifications (5-mCyt and 5-hmCyt) on DNA mismatch repair and the long-path BER-dependent pathway^32^.

It must be stressed that the fruit fly possesses orthologs of human SMUG1 (CG5825), TDG (Thd1), and MBD4 (MBD-R2). Furthermore, the fruit fly lacks a DNA polymerase β ortholog, similar to other *Diptera*, which suggests that *Diptera* use only long-patch BER^33^.

Vitamin C (L-ascorbic acid) is a known activator of proteins from the family of Fe^2+^ and 2-oxyglutarate-dependent dioxygenases, which include TET proteins^34^. It was shown that L-AA may enhance the generation of DNA modification both in vitro and in vivo^35–38^. The results of our research on the HCT 116 cell line^39^ and other human cell lines^40^ confirm an increase in the levels of 5-hmCyt, 5-fCyt, 5-caCyt and 5-hmUra in cells supplemented with L-AA. For this reason, we decided to check whether these trends applied only to mammalian cells or whether a similar effect would be obtained in the case of *Drosophila* S2 cells. It was not possible to determine the 5-hmCyt level in S2 cells because its content was at the border of the quantitative detection of the applied method. A time-dependent increase in 5-hmUra content in cells cultured with the addition of 1 mM L-AA (Figs. 4, 5, 6) was noted. It is worth noting that the maximum concentration of L-AA in the supplemented cell medium (~ 0.555 mM, Fig. 7) was similar to the average concentration of L-AA observed in larvae, pupae and adult flies (Supplementary Fig. S1). The presented reports and our results may be seen in the context of dTet functions. The *D. melanogaster* genome contains relatively low levels of 5-mCyt, but it is readily observed in the fly genome. It is possible that in early developmental stages, 5-hmUra plays a significant role in removing 5-mCyt^30,31^ to activate certain genes, as described above. Our results suggest that a certain pool of 5-hmUra observed in *D. melanogaster* may arise from the enzymatic activity of dTET protein. Interestingly, the levels of 5-hmUra in larval and pupal DNA were significantly higher than those observed in human cells^41,42^, which suggests that this modification plays an important role during *D. melanogaster* development.

Interestingly, although *D. melanogaster* does not have L-gulonolactone oxidase (GULO, the enzyme responsible for the last stage of L-ascorbate synthesis), this vitamin plays an important role^43^. L-AA supplementation was found to affect the lifespan of flies^44^. Henriques et al. found that *Drosophila* is capable of synthesizing L-AA, likely through an alternative pathway^43^. In our study, the level of L-AA in *D. melanogaster* was determined. The obtained concentrations in a single cell reached 0.53 mM (see Supplementary Fig. S1), a level similar to those reported for some animal tissues^45^. Interestingly, at this concentration, the highest level of 5-hmUra in *Drosophila* cell culture was recognized (Fig. 6).

In 2015, the presence of N6-mAde in *D. melanogaster* DNA was confirmed. The abundance of this modification appeared to display a peak (∼0.07%, N6-mAde/Ade) in ∼0.75 h embryos but was dramatically reduced to a very low level (∼0.001%, N6-mAde/Ade) in 4–16 h embryos. Additionally, the level of N6-mAde in adult tissues (brain and ovary) was quantified, and it exhibited similar low levels to those found in the late-stage embryonic genome^16^. For this reason, we decided to extend the panel of the analyzed DNA modifications with N6-mAde. The highest levels were observed in larvae 1–2 days after hatching and in imagos 1 day after eclosion. The lowest level of this modification was found in 5-day-old and 20-day-old imagos. However, recent data suggest that the previously reported levels of N6-mAde in the fruit fly are significantly overstated. Recently, a reliable 6mASCOPE method was used. Kong et al. described in Science that the level of this modification in embryos and adult flies was ~ 0.0002% N6-mAde/Ade^46^. Our analyses of 5- and 20-day-old imago DNA are in good agreement with the aforementioned studies.

The level of DNA modification was also compared between insects bred in the optimal temperature conditions (25 °C) and at 18 °C. The lower bred temperature resulted in a dramatic increase in the level of N6-mAde (Fig. 3d). This rise is likely to represent a regulatory/response role postulated for N6-mAde^47^. The lower temperature also led to a significant decrease in the 5-hmUra level (Fig. 2d). This, in turn, should suppress the processive demethylation pathway and, as a consequence, be responsible for the observed rise in the 5-mCyt and 5-hmCyt levels (Fig. 1b,d).

In conclusion, our results suggest that the profound changes in N6-mAde and 5-hmUra levels during the fruit fly life cycle and as a result of bred temperature changes indicate that these DNA modifications play important regulatory roles in response to environmental changes and/or biological conditions.

Here, we presented, for the first time, a broad analysis of the levels of DNA modifications in the genetic material from various developmental stages of *D. melanogaster* (larvae, pupae, adult flies). Moreover, it was possible to observe significant differences between groups of insects bred under different temperature conditions (different metabolic rates). It was also shown that it is possible to induce changes in the content of epigenetic derivatives in *Drosophila* Schneider 2 cells—an in vitro model of *D. melanogaster*. The obtained results suggest that each of the analyzed modifications may play a role as a potential epigenetic DNA marker in *D. melanogaster*.

## Material and methods

### *Drosophila* handling procedures and synchronized culture

The wild-type *D. melanogaster* population used in this study originated from two natural populations in Bydgoszcz, Poland (GPS coordinates: 53° 11.8′ N 17° 56.9′ E and 53° 10.5′ N 18° 08.7′ E). The flies from those two populations were mass crossed and bred for a maximum of 10–12 generations. The experimental insects were cultivated on a standard medium containing agar, yeast, banana, and sugar with the addition of antibiotics (tetracycline and ampicillin) and antifungal agents (p-hydroxy-benzoic acid methyl ester in ethanol and phosphoric + propionic acid mix) (details in Supplementary Table S15). The incubator provided a 12 h light/dark cycle and a humidity of ~ 65%. The experiments were carried out on two groups of insects bred under different temperature conditions (in the optimal conditions (25 °C) and at 18 °C).

To synchronize breeding, adult flies were placed on fresh medium and left for 24 h until eggs were laid; next, the imagos were removed from the media. As part of the experiments, individuals from 3 developmental stages were collected: larvae, pupae and imagos (details in Table 1). Ether anesthesia was used for adult flies. The obtained samples were immediately frozen at − 80 °C and stored until analysis.

**Table 1:** Characteristics of the studied groups.

Breeding at 25 °C		Breeding at 18 °C	
Larvae 1	Larvae 1–2 days after hatching	Larvae 3	Larvae 3–4 days after hatching
Larvae 4	Larvae ~ 4 days after hatching	Larvae 10	Larvae ~ 10 days after hatching
Pupae 1	Pupae ~ 24 h after metamorphosis	Pupae 1	Pupae 1–2 days after metamorphosis
		Pupae 3	Pupae 3–4 days after metamorphosis
Pupae 3	3 days old pupae (just before turning into imago)	Pupae 6	6–7 days old pupae (just before turning into imago)
Imago 1	Imago 1 day after eclosion	Imago 1	Imago 1 day after eclosion
Imago 5	Imago 5 days after eclosion	Imago 5	Imago 5 days after eclosion
Imago 20	Imago 20 days after eclosion	Imago 20	Imago 20 days after eclosion

### Culture of the *Drosophila* Schneider 2 cell line

*Drosophila* S2 cells (Thermo Fisher, Cat. No. R69007) were cultured in accordance with the manufacturers’ guidelines. Namely, frozen cells were thawed in a 30 °C water bath and immediately transferred to 15 mL polypropylene tubes containing 10 mL of Schneider's *Drosophila* Medium (Biological Industries) supplemented with 10% heat-inactivated FBS (PAN-Biotech) and 1% antibiotic–antimycotic solution (Corning) preheated to 27 °C. The cell suspension was centrifuged (5 min, 200 × g, 27 °C), the supernatant containing DMSO was discarded, and the cells were resuspended in 1 mL of complete growth medium and counted in a LUNA-II Automated Cell Counter (Logos Biosystems) to assess the cell density and viability. Next, approximately 2 × 10^6^ cells/mL were seeded in 25 cm^2^ culture flasks (Thermo Fisher) and incubated in a 27 °C nonhumidified, ambient air-regulated incubator. After reaching a density of 2 × 10^7^ cells/mL, the cells were split at a 1:5 dilution ratio into new culture vessels. Cells from passages 6–10 were used in the experiments described in Supplementary Table S16. In each experiment, a 100 mM stock solution of L-AA in Dulbecco's phosphate-buffered saline (DPBS) was freshly prepared and added to the culture medium to the desired final concentration. Control cells were treated with a volumetric equivalent of DPBS. After the incubation time described in each experiment, cells were harvested by scraping the surface of the culture vessel with a cell scraper and pipetting up and down the solution to break up clumps of cells. Subsequently, the cell suspension was transferred to 15 mL polypropylene tubes and centrifuged for 5 min (200 × g, 27 °C). The obtained cell pellets were frozen and stored at – 80 °C and subsequently used in downstream analyses.

### DNA isolation from larvae, pupae and adult flies

The method of isolating the genetic material from adult flies was described by Starczak et al.^48^. In this study, the method was subtly altered to obtain the genetic material from all the analyzed stages of *D. melanogaster*. Briefly, 1 mL of ice-cold *Drosophila* buffer A (200 mM Tris–HCl, 60 mM NaCl, 10 mM Na_2_EDTA, 0.5% Triton X-100, 100 mM L-lysine; pH 9.4) was added to the samples (larvae 100–200 mg; pupae 60–100 mg; imago 20–60 mg). The insects were homogenized with a stainless steel pestle for approximately 3 min on ice and then centrifuged for 2 min at 1875 × g (horizontal rotor) at 4 °C. The supernatant was transferred to a clean tube, and 600 µL of *Drosophila* buffer B (200 mM Tris–HCl, 60 mM NaCl, 10 mM Na_2_EDTA, 0.5% Triton X-100; pH 9.2) was added. Next, the samples were centrifuged for 15 min at 1600 × g (fixed angle rotor) at 4 °C. The obtained pellet was suspended in 500 µL of *Drosophila* buffer B and centrifuged under the same conditions. The pellet was suspended in 350 µL of *Drosophila* buffer B, 60 µL of RNase A (2 mg/mL, Sigma), 20 µL of RNase T1 (2 U/µL, Sigma) and 300 µL of 10% SDS were added, and the mixture was gently mixed. The samples were incubated at 37 °C for 30 min. Proteinase K was added to a final concentration of 4 mg/mL and incubated at 37 °C for 1.5 h. The mixture was transferred to a plastic tube with 700 µL of phenol:chloroform:isoamyl alcohol (25:24:1), vortexed vigorously and centrifuged for 15 min at 2800 × g at 4 °C. After the extraction, the aqueous phase was treated with one volume of chloroform:isoamyl alcohol mixture (24:1) and centrifuged under the same conditions. The supernatant was treated with three volumes of cold 96% (v/v) ethanol to precipitate high molecular weight nucleic acids. The precipitate was removed with a plastic spatula, washed with ethanol and dissolved in 50 µL of Milli-Q grade deionized water.

### DNA extraction from Schneider 2 cells

DNA extraction from cells was performed according to a previously published protocol^48^ (details are provided in the Supplementary Materials).

### DNA hydrolysis to deoxynucleosides

DNA samples were treated with 100 U of nuclease P1 (New England Biolabs) for 3 h in a buffer containing 200 mM ammonium acetate, 0.2 mM ZnCl_2_ (pH 4.6) and 10 μg/sample tetrahydrouridine at 37 °C, followed by the addition of 10% NH_4_OH and 12 U or 6 U (for the DNA from insects and S2 cells, respectively) of shrimp alkaline phosphatase (New England Biolabs) and a subsequent additional 1.5 h incubation at 37 °C. Finally, all the hydrolysates were ultrafiltered prior to injection and concentrated in a vacuum concentrator (to a final volume of ~ 20 μL). The DNA hydrolysates were spiked with a mixture of internal standards at a volumetric ratio of 4:1 to a concentration of 50 fmol/µL [D_3_]-5-hmdC, [^13^C_10_, ^15^N_2_]-5-mdC, [^13^C_10_, ^15^N_2_]-5-formyl-2′-deoxycytidine, [^13^C_10_, ^15^N_2_]-5-carboxy-2′-deoxycytidine, [^13^C_10_, ^15^N_2_]-5-hmdU, [^13^C_10_, ^15^N_2_]-dU, and [^15^N_5_]-8-oxodG and 5 fmol/µL [D_3_]-N6-mdA.

### Mass spectrometry profiling of modified nucleotides

Analyses were performed using a method described earlier by Gackowski et al.^49^ with some modifications (details are provided in the Supplementary Materials).

### Analysis of L-ascorbic acid levels in cells and culture medium

To analyze the concentration of L-AA, both in the culture medium and inside the cells, the previously described ultra-performance liquid chromatography (UPLC) with the UV detection method was used^39^ (details are provided in the Supplementary Materials).

### Determination of intracellular concentrations of ascorbate in *Drosophila melanogaster*

Approximately 10 mg of samples with 200 µL of PBS were homogenized with a BEAD RUPTOR ELITE Bead Mill Homogenizer (OMNI International) using 1.4 mm ceramic bead media (OMNI International). Subsequently, the samples were homogenized with an ultrasonic homogenizer (SONOPULS UW 2070, BANDELIN electronic GmbH&Co. KG) two times for 10 s on ice. Forty-five microliters of sample was mixed with 50 µL of 10% trichloroacetic acid and 5 µL of 100 µM labeled internal standard solution (L-ascorbic acid-^13^C_6_, Toronto Research Chemicals) and incubated for 20 min. Then, the samples were vortexed and centrifuged at 24 400 × g for 20 min at 4 °C. The supernatants were filtrated using AcroPrep Advance 96-Well Filter Plates 10 K. One microliter of the aliquots were chromatographically separated on a CORTECS UPLC T3 1.6 µm (2.1 mm × 100 mm) column with a Waters Xevo TQ-XS tandem mass spectrometer. The column (20 °C) was eluted at a flow rate of 0.3 mL/min with 5 µM ammonium formate in 0.05% acetic acid (solvent A) and methanol (solvent B) (details in Supplementary Table S18). The electrospray ionization was set to negative ion mode. The desolvation gas (nitrogen) flow rate was 1200 L/h, the nitrogen cone gas flow was 200 L/h, the desolvation temperature was 500 °C and the nebulizer gas pressure was 7 bar. Collision-induced dissociation was obtained with argon (6.0 at 3 × 10^-6^ bar pressure) as the collision gas. Transition patterns that were selected as quantitative (175 > 115 and 181 > 119 for L-ascorbic acid and [^13^C_6_]-L-ascorbic acid, respectively) were acquired using MassLynx 4.2 software from Waters. Quantitative analyses were performed using the Target Lynx application. All the samples were analyzed in three to five technical replicates.

### Determination of thymine in acidic hydrolysates of *Drosophila* extracts

The determination of thymine in homogenates from three developmental stages of *D. melanogaster* (larvae, pupae, imago) was performed using the method described by Modrzejewska et al.^50^ with some modifications (details are provided in the Supplementary Materials).

### Statistical analysis

The results of the experiments carried out on the in vitro model are presented as the mean, mean ± standard error and mean ± standard deviation. One-way analysis of variance (ANOVA) with LSD and Tukey’s HSD post hoc test were used to verify the significance of differences between cells cultured in various conditions. The results of experiments comparing the developmental stages of *D. melanogaster* are presented as the mean and median values, interquartile ranges, and nonoutlier ranges. The nonparametric Kruskal‒Wallis test was used for between-group comparisons. Statistical analyses were carried out with Statistica 13.1 PL software (TIBCO Software Inc. (2017) Statistica (data analysis software system), version 13. http://statistica.io.). The results were considered statistically significant at *p* values less than 0.05.

### Ethics declaration

In accordance with Polish law, experiments using invertebrates do not require the consent of the bioethics committee.

## Supplementary Information

Supplementary Information.

## Data Availability

The datasets generated and/or analyzed during the current study are available in the RepOD repository, [10.18150/Y2ANHC].
