# Supplementary material for: Dynamic changes in genomic 5-hydroxymethyluracil and N6-methyladenine levels in the Drosophila melanogaster life cycle and in response to different temperature conditions
Source: Sci Rep. 2022 Oct 20;12:17552. doi: 10.1038/s41598-022-22490-9 (PMC9584883; doi:10.1038/s41598-022-22490-9)
Supplement: Supplementary file 1 — Supplementary Information. [file 41598_2022_22490_MOESM1_ESM.pdf]

Dynamic changes in genomic 5-hydroxymethyluracil and N6-methyladenine levels in the Drosophila melanogaster life cycle and in response to different temperature conditions

Marta Starczak[1,*], Maciej Gawronski[1], Aleksandra Wasilow[1], Pawel Mijewski[1], Ryszard Olinski[1], Daniel Gackowski[1,*]

[1] Department of Clinical Biochemistry, Faculty of Pharmacy, Collegium Medicum in Bydgoszcz, Nicolaus Copernicus University in Toruń, 85-092, Bydgoszcz, Poland.

[*] Corresponding authors

Supplementary Materials

Supplementary Table S1 Comparison of the levels of potential epigenetic DNA modifications in the development stages of *Drosophila melanogaster* bred at 25 ˚C.

Supplementary Table S2 Comparison of the levels of potential epigenetic DNA modifications in the development stages of *Drosophila melanogaster* bred at 18 ˚C.

Supplementary Table S3 Results of Kruskal-Wallis test for comparisons of levels of DNA modifications between breeding temperatures (same developmental stage).

Supplementary Table S4 Results of Kruskal-Wallis test for comparisons of levels of 5-methyl-2’-deoxycytidine and 5-(hydroxymethyl)-2’-deoxycytidine in developmental stages of *Drosophila melanogaster* bred under different temperature conditions.

Supplementary Table S5 Results of Kruskal-Wallis test for comparisons of levels of 2’-deoxyuridine and 5-(hydroxymethyl)-2’-deoxyuridine in developmental stages of *Drosophila melanogaster* bred under different temperature conditions.

Supplementary Table S6 Results of Kruskal-Wallis test for comparisons of levels of 8-oxo-7,8-dihydro-2’-deoxyguanosine and N6-methyl-2’-deoxyadenosine in developmental stages of *Drosophila melanogaster* bred under different temperature conditions.

Supplementary Figure S1 L-ascorbic acid (L-AA) concentration in *D. melanogaster* larvae, pupae and imago.

Supplementary Table S7 The levels of potential epigenetic DNA modifications in S2 cells cultured at medium supplemented with different concentration of L-ascorbic acid.

Supplementary Table S8 Results of post-hoc test for comparisons of levels of DNA modifications in DNA of S2 cells after 72-hour cultivation in the presence of different concentrations of L-ascorbic acid.

Supplementary Table S9 The levels of potential epigenetic DNA modifications in S2 cells cultured with addition of 1 mM L-AA for 24h, 48h and 72h.

Supplementary Table S10 Results of post-hoc test for comparisons of levels of DNA modifications in DNA of S2 cells after culture with 1 mM L-ascorbic acid for 24h, 48h and 72h.

Supplementary Table S11 Kinetics of DNA modification in S2 cells supplemented with 1 mM L-ascorbic acid and in control cells.

Supplementary Table S12 Results of post-hoc test for comparisons of level of 5-(hydroxymethyl)-2'-deoxyuridine in DNA of S2 cells supplemented with 1 mM L-ascorbic acid.

Supplementary Table S13 Results of post-hoc test for comparisons of level of 2'-deoxyuridine in DNA of S2 cells supplemented with 1 mM L-ascorbic acid.

Supplementary Table S14 Results of post-hoc test for comparisons of level of 8-oxo-7,8-dihydro-2'-deoxyguanosine in DNA of S2 cells supplemented with 1 mM L-ascorbic acid.

Supplementary Figure S2 Correlation between the level of 8-oxo-7,8-dihydro-2'-deoxyguanosine and 5-(hydroxymethyl)-2'-deoxyuridine.

Supplementary Table S15 *Drosophila* medium recipe.

Supplementary Table S16 Description of experiments on *Drosophila* S2 cells.

Supplementary Methods: DNA extraction from Schneider 2 cells; Mass-spectrometry profiling of modified nucleotides.

Supplementary Table S17 Transition patterns-specific detector settings and sources of standards for the analysed compounds.

Supplementary Methods: Analysis of L-ascorbic acid levels in cells and culture medium

Supplementary Table S18 Flow gradient used for elution during determination of intracellular L-AA concentration in *D. melanogaster*.

Supplementary Methods: Determination of thymine in acidic hydrolysates of *Drosophila* extracts.

Supplementary References

	modifications per 10^6 dN					
	**5-methyl-2'-deoxycytidine**	**5-(hydroxymethyl)-2'-deoxycytidine**	**2'-deoxyuridine**	**5-(hydroxymethyl)-2'-deoxyuridine**	**8-oxo-7,8-dihydro-2'-deoxyguanosine**	**N6-methyl-2'-deoxyadenosine**
	mean±SD	mean±SD	mean±SD	mean±SD	mean±SD	mean±SD
	median (interquartile range)	median (interquartile range)	median (interquartile range)	median (interquartile range)	median (interquartile range)	median (interquartile range)
**larvae 1**	79.377±45.831	0.041±0.016	13.705±2.494	6.100±2.191	1.126±0.442	41.780±32.467
	68.735 (45.247;101.664)	0.034 (0.029;0.053)	12.860 (12.356;13.776)	6.422 (4.337;7.737)	0.902 (0.875;1.230)	37.517 (16.921;71.755)
**larvae 4**	35.621±16.536	0.072±0.018	17.062±2.434	2.056±0.892	2.139±0.990	15.049±8.872
	29.569 (24.272;46.971)	0.070 (0.061;0.084)	16.595 (15.018;18.238)	1.979 (1.157;3.123)	1.753 (1.438;2.534)	12.013 (8.614;24.712)
**pupae 1**	18.646±9.396	0.077±0.018	12.531±1.374	3.676±1.659	1.293±0.164	27.858±17.572
	16.505 (15.707;25.080)	0.077 (0.057;0.091)	12.226 (11.692;13.836)	3.930 (2.286;4.717)	1.306 (1.146;1.400)	22.709 (16.081;29.298)
**pupae 3**	15.546±3.326	0.071±0.020	11.737±0.724	1.297±0.452	1.396±0.437	24.342±6.521
	15.474 (12.926;17.118)	0.069 (0.053;0.084)	11.699 (11.179;12.025)	1.172 (0.936;1.447)	1.293 (1.170;1.373)	23.803 (19.962;28.924)
**imago 1**	19.863±6.546	0.078±0.038	11.606±0.742	1.179±0.536	1.691±0.605	46.496±45.571
	20.664 (14.001;23.708)	0.082 (0.040;0.109)	11.549 (10.938;12.285)	0.950 (0.742;1.583)	1.573 (1.343;1.921)	50.010 (1.666;60.589)
**imago 5**	21.167±6.787	0.081±0.018	12.782±1.006	3.776±1.851	1.813±0.465	4.172±2.618
	17.806 (16.955;24.027)	0.075 (0.069;0.093)	12.494 (12.007;13.268)	4.104 (2.902;5.588)	1.815 (1.409;2.250)	3.400 (2.533;5.731)
**imago 20**	16.703±12.491	0.120±0.046	14.126±1.452	3.677±1.193	1.816±0.644	5.971±6.079
	10.732 (7.491;24.422)	0.137 (0.091;0.157)	13.966 (13.066;15.194)	3.500 (2.909;3.874)	1.863 (1.183;2.182)	4.215 (2.671;6.515)

Supplementary Table S1 Comparison of the levels of potential epigenetic DNA modifications in the development stages of *Drosophila melanogaster* bred at 25 ˚C.

	modifications per 10^6 dN					
	**5-methyl-2'-deoxycytidine**	**5-(hydroxymethyl)-2'-deoxycytidine**	**2'-deoxyuridine**	**5-(hydroxymethyl)-2'-deoxyuridine**	**8-oxo-7,8-dihydro-2'-deoxyguanosine**	**N6-methyl-2'-deoxyadenosine**
	mean±SD	mean±SD	mean±SD	mean±SD	mean±SD	mean±SD
	median (interquartile range)	median (interquartile range)	median (interquartile range)	median (interquartile range)	median (interquartile range)	median (interquartile range)
**larvae 3**	111.085±122.901	0.201±0.055	15.882±1.429	1.290±0.244	2.608±0.663	185.668±45.533
	56.985 (37.577;172.225)	0.183 (0.156;0.217)	15.574 (15.062;16.795)	1.239 (1.069;1.518)	2.367 (2.220;3.218)	187.524 (135.131;208.097)
**larvae 10**	36.026±6.839	0.183±0.029	16.938±1.737	1.202±0.242	2.579±0.659	106.324±19.507
	33.212 (32.089;39.979)	0.194 (0.162;0.204)	16.655 (16.476;18.575)	1.149 (1.050;1.421)	2.495 (1.969;3.146)	97.520 (93.380;121.954)
**pupae 1**	79.733±30.519	0.153±0.045	14.675±0.540	1.523±0.230	1.945±0.321	152.463±21.538
	73.056 (57.251;92.053)	0.136 (0.130;0.163)	14.911 (14.068;15.114)	1.501 (1.444;1.696)	1.876 (1.699;2.070)	147.309 (136.771;175.861)
**pupae 3**	438.128±95.004	0.202±0.050	13.936±1.166	1.771±0.452	1.912±0.232	199.562±22.003
	458.963 (335.424;512.412)	0.181 (0.178;0.201)	13.846 (13.207;14.609)	1.763 (1.661;1.882)	1.846 (1.758;2.161)	197.877 (183.444;210.569)
**pupae 6**	171.753±122.850	0.290±0.198	12.798±0.758	2.089±0.386	1.871±0.155	353.613±59.438
	110.562 (94.710;227.775)	0.216 (0.143;0.395)	12.892 (12.255;13.478)	2.144 (1.707;2.359)	1.864 (1.795;1.938)	352.650 (294.358;396.219)
**imago 1**	54.829±25.389	0.150±0.032	13.584±0.494	1.498±0.172	2.134±0.260	191.476±33.841
	48.297 (41.704;51.921)	0.151 (0.127;0.183)	13.485 (13.175;13.941)	1.459 (1.369;1.594)	2.048 (1.953;2.187)	174.828 (166.947;220.074)
**imago 5**	44.542±11.415	0.119±0.032	14.390±2.129	1.389±0.280	1.803±0.086	198.787±95.061
	41.541 (33.307;53.756)	0.126 (0.091;0.147)	13.144 (12.844;16.308)	1.534 (1.105;1.624)	1.796 (1.769;1.821)	192.845 (103.810;275.510)
**imago 20**	31.880±7.142	0.133±0.061	14.655±0.941	1.340±0.332	2.092±0.259	107.356±23.000
	30.859 (25.844;39.698)	0.101 (0.086;0.179)	14.716 (14.105;14.984)	1.229 (1.121;1.529)	2.079 (2.005;2.215)	111.590 (85.502;129.772)

Supplementary Table S2 Comparison of the levels of potential epigenetic DNA modifications in the development stages of *Drosophila melanogaster* bred at 18 ˚C.

	larvae 1 vs. larvae 3	larvae 4 vs. larvae 10	pupae1 (25 °C) vs. pupae1 (18 °C)	pupae 3 vs. pupae 6	imago1 (25 °C) vs. imago 1 (18 °C)	imago5 (25 °C) vs. imago 5 (18 °C)	imago20 (25 °C) vs. imago 20 (18 °C)
**5-methyl-2’-deoxycytidine**	1.0000	1.0000	**0.0067**	**0.0004**	0.8057	1.0000	1.0000
**5-(hydroxymethyl)-2’-deoxycytidine**	**0.0003**	**0.0022**	**0.0002**	**0.0011**	**0.0009**	**0.0248**	1.0000
**2’-deoxyuridine**	**0.0105**	0.9025	**0.0015**	**0.0227**	**0.0002**	0.0637	0.3525
**5-(hydroxymethyl)-2’-deoxyuridine**	**0.0003**	0.0662	**0.0012**	**0.0143**	0.1509	**0.0192**	**0.0004**
**8-oxo-7,8-dihydro-2’-deoxyguanosine**	**0.0005**	0.1416	**0.0002**	**0.0143**	**0.0193**	0.9136	0.2719

Supplementary Table S3 Results of Kruskal-Wallis test for comparisons of levels of DNA modifications between breeding temperatures (same developmental stage).

5-methyl-2’-deoxycytidine (25˚C)							
	**imago 20**	**imago 5**	**imago1**	**pupae 3**		**pupae 1**	**larvae 4**
**larvae 1**	**0.0004**	0.1896	0.1650	**0.0011**		**0.0241**	1.0000
**larvae 4**	0.2418	1.0000	1.0000	0.3578		1.0000	
**pupae 1**	1.0000	1.0000	1.0000	1.0000			
**pupae3**	1.0000	1.0000	1.0000				
**imago 1**	1.0000	1.0000					
**imago 5**	1.0000						

5-(hydroxymethyl)-2’-deoxycytidine (25˚C)							
	**imago 20**	**imago 5**	**imago1**	**pupae 3**		**pupae 1**	**larvae4**
**larvae 1**	**0.0002**	0.1193	0.2064	1.0000		0.2292	0.9215
**larvae 4**	0.3703	1.0000	1.0000	1.0000		1.0000	
**pupae 1**	1.0000	1.0000	1.0000	1.0000			
**pupae3**	0.2841	1.0000	1.0000				
**imago 1**	1.0000	1.0000					
**imago 5**	1.0000						

5-methyl-2’-deoxycytidine (18˚C)							
	**imago 20**	**imago 5**	**imago1**	**pupae 3**	**pupae 6**	**pupae 1**	**larvae 10**
**larvae 3**	0.3784	1.0000	1.0000	1.0000	**0.0219**	1.0000	1.0000
**larvae 10**	1.0000	1.0000	1.0000	0.0520	**0.0002**	0.3284	
**pupae 1**	**0.0244**	1.0000	1.0000	1.0000	0.4436		
**pupae 3**	**<0.0001**	**0.0015**	**0.0032**	1.0000			
**pupae 6**	**0.0032**	0.3009	0.7286				
**imago 1**	1.0000	1.0000					
**imago 5**	1.0000						

5-(hydroxymethyl)-2’-deoxycytidine (18˚C)							
	**imago 20**	**imago 5**	**imago1**	**pupae 3**	**pupae 6**	**pupae 1**	**larvae 10**
**larvae 3**	0.1622	**0.0273**	1.0000	1.0000	1.0000	0.4368	1.0000
**larvae 10**	1.0000	0.3367	1.0000	1.0000	1.0000	1.0000	
**pupae 1**	1.0000	1.0000	1.0000	1.0000	0.4043		
**pupae 3**	0.1507	**0.0260**	1.0000	1.0000			
**pupae 6**	0.4253	0.1058	1.0000				
**imago 1**	1.0000	1.0000					
**imago 5**	1.0000						

Supplementary Table S4 Results of Kruskal-Wallis test for comparisons of levels of 5-methyl-2’-deoxycytidine and 5-(hydroxymethyl)-2’-deoxycytidine in developmental stages of *Drosophila melanogaster* bred under different temperature conditions.

2’-deoxyuridine (25˚C)							
	**imago 20**	**imago 5**	**imago1**	**pupae 3**		**pupae 1**	**larvae 4**
**larvae 1**	1.0000	1.0000	0.3537	0.6390		1.0000	0.4780
**larvae 4**	1.0000	**0.0346**	**<0.0001**	**<0.0001**		**0.0052**	
**pupae 1**	0.7948	1.0000	1.0000	1.0000			
**pupae3**	**0.0154**	1.0000	1.0000				
**imago 1**	**0.0062**	1.0000					
**imago 5**	1.0000						

5-(hydroxymethyl)-2’-deoxyuridine (25˚C)							
	**imago 20**	**imago 5**	**imago1**	**pupae 3**		**pupae 1**	**larvae4**
**larvae 1**	1.0000	1.0000	**<0.0001**	**0.0001**		1.0000	**0.0205**
**larvae 4**	0.8967	1.0000	1.0000	1.0000		1.0000	
**pupae 1**	1.0000	1.0000	**0.0123**	**0.0388**			
**pupae3**	**0.0149**	0.0586	1.0000				
**imago 1**	**0.0041**	**0.0192**					
**imago 5**	1.0000						

2’-deoxyuridine (18˚C)							
	**imago 20**	**imago 5**	**imago1**	**pupae 3**	**pupae 6**	**pupae 1**	**larvae 10**
**larvae 3**	1.0000	0.2937	**0.0047**	**0.0008**	0.1103	1.0000	1.0000
**larvae 10**	1.0000	0.2646	**0.0121**	**0.0020**	0.1365	1.0000	
**pupae 1**	1.0000	1.0000	0.7528	0.1284	1.0000		
**pupae 3**	1.0000	1.0000	1.0000	1.0000			
**pupae 6**	0.3805	1.0000	1.0000				
**imago 1**	1.0000	1.0000					
**imago 5**	1.0000						

5-(hydroxymethyl)-2’-deoxyuridine (18˚C)							
	**imago 20**	**imago 5**	**imago1**	**pupae 3**	**pupae 6**	**pupae 1**	**larvae 10**
**larvae 3**	1.0000	1.0000	1.0000	**0.0115**	0.0536	1.0000	1.0000
**larvae 10**	1.0000	1.0000	1.0000	**0.0160**	0.0823	1.0000	
**pupae 1**	1.0000	1.0000	1.0000	1.0000	1.0000		
**pupae 3**	0.3487	1.0000	1.0000	1.0000			
**pupae 6**	0.0657	0.2679	0.7380				
**imago 1**	1.0000	1.0000					
**imago 5**	1.0000						

Supplementary Table S5 Results of Kruskal-Wallis test for comparisons of levels of 2’-deoxyuridine and 5-(hydroxymethyl)-2’-deoxyuridine in developmental stages of *Drosophila melanogaster* bred under different temperature conditions.

8-oxo-7,8-dihydro-2’-deoxyguanosine (25°C)							
	**imago 20**	**imago 5**	**imago1**	**pupae 3**		**pupae 1**	**larvae 4**
**larvae 1**	0.0843	**0.0365**	0.2620	1.0000		1.0000	**0.0095**
**larvae 4**	1.0000	1.0000	1.0000	0.6069		0.3093	
**pupae 1**	1.0000	0.8811	1.0000	1.0000			
**pupae3**	1.0000	1.0000	1.0000				
**imago 1**	1.0000	1.0000					
**imago 5**	1.0000						

N6-methyl-2’-deoxyadenosine (25°C)							
	**imago 20**	**imago 5**	**imago1**	**pupae 3**		**pupae 1**	**larvae4**
**larvae 1**	**0.0448**	**0.0352**	1.0000	1.0000		1.0000	1.0000
**larvae 4**	1.0000	1.0000	1.0000	1.0000		1.0000	
**pupae 1**	**0.0281**	**0.0229**	1.0000	1.0000			
**pupae3**	**0.0320**	**0.0257**	1.0000				
**imago 1**	0.0865	0.0683					
**imago 5**	1.0000						

8-oxo-7,8-dihydro-2’-deoxyguanosine (18°C)							
	**imago 20**	**imago 5**	**imago1**	**pupae 3**	**pupae 6**	**pupae 1**	**larvae 10**
**larvae 3**	1.0000	**0.0241**	1.0000	0.2870	0.1140	0.1169	1.0000
**larvae 10**	1.0000	0.1721	1.0000	0.9096	0.6725	0.7278	
**pupae 1**	1.0000	1.0000	1.0000	1.0000	1.0000		
**pupae 3**	1.0000	1.0000	1.0000	1.0000			
**pupae 6**	1.0000	1.0000	1.0000				
**imago 1**	1.0000	0.7619					
**imago 5**	1.0000						

N6-methyl-2’-deoxyadenosine (18°C)							
	**imago 20**	**imago 5**	**imago1**	**pupae 3**	**pupae 6**	**pupae 1**	**larvae 10**
**larvae 3**	0.0769	1.0000	1.0000	0.1942	1.0000	1.0000	0.2086
**larvae 10**	1.0000	0.5028	0.1429	**0.0001**	**0.0308**	1.0000	
**pupae 1**	1.0000	1.0000	1.0000	**0.0052**	1.0000		
**pupae 3**	**0.0077**	1.0000	1.0000	1.0000			
**pupae 6**	**<0.0001**	0.2875	0.4915				
**imago 1**	0.0527	1.0000					
**imago 5**	0.2604						

Supplementary Table S6 Results of Kruskal-Wallis test for comparisons of levels of 8-oxo-7,8-dihydro-2’-deoxyguanosine and N6-methyl-2’-deoxyadenosine in developmental stages of *Drosophila melanogaster* bred under different temperature conditions.

Supplementary Figure S1 L-ascorbic acid (L-AA) concentration in *D. melanogaster* larvae, pupae and imago.

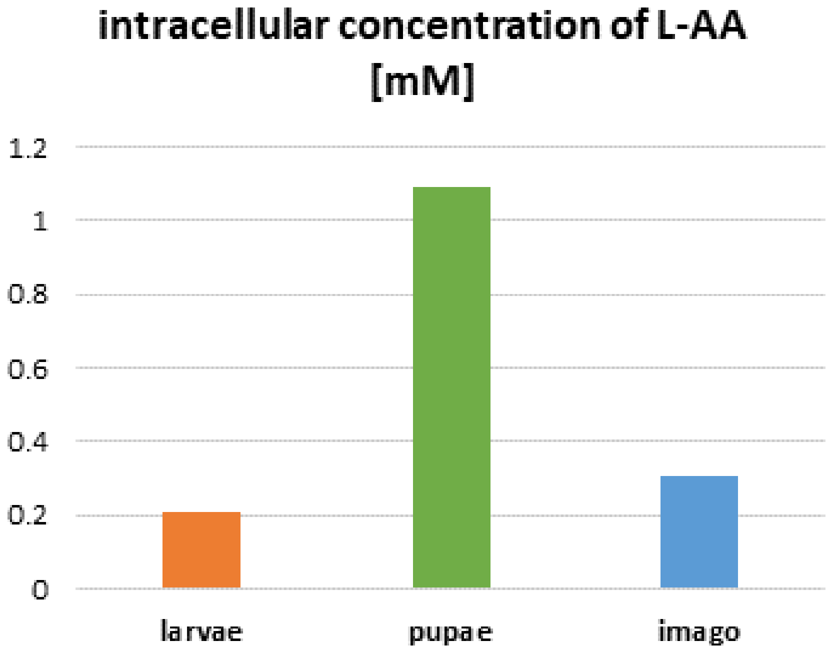

	larvae	pupae	imago	mean
**Intracellular concentration of L-AA [mM]**	0.2067	1.0925	0.3047	0.5346

	**modifications per 10^6 dN**			
	**5-methyl-2'-deoxycytidine**	**5-(hydroxymethyl)-2'-deoxyuridine**	**2'-deoxyuridine**	**8-oxo-7,8-dihydro-2'-deoxyguanosine**
	mean±SD	mean±SD	mean±SD	mean±SD
	median (interquartile range)	median (interquartile range)	median (interquartile range)	median (interquartile range)
**control 72h**	0.200±0.039	2.241±0.107	8.685±0.413	1.074±0.088
	0.181 (0.174;0.245)	2.227 (2.142;2.355)	8.649 (8.291;9.115)	1.088 (0.979;1.153)
**10 µM L-AA 72h**	0.205±0.026	2.234±0.071	8.389±0.253	1.160±0.096
	0.206 (0.178;0.230)	2.268 (2.152;2.282)	8.515 (8.097;8.554)	1.110 (1.099;1.271)
**100 µM L-AA 72h**	0.305±0.147	2.454±0.244	8.693±0.361	0.992±0.045
	0.224 (0.217;0.474)	2.516 (2.186;2.661)	8.776 (8.298;9.006)	0.986 (0.949;1.040)
**1 mM L-AA 72h**	0.334±0.091	11.482±0.506	8.370±0.040	1.298±0.158
	0.336 (0.241;0.424)	11.225 (11.157;12.065)	8.388 (8.325;8.397)	1.304 (1.138;1.453)

Supplementary Table S7 The levels of potential epigenetic DNA modifications in S2 cells cultured at medium supplemented with different concentration of L-ascorbic acid.

5-methyl-2'-deoxycytidine			
	**1mM L-AA 72h**	**100 µM L-AA 72h**	**10 µM L-AA 72h**
**Control 72h**	0.3291	0.5134	0.9999
**10 µM L-AA 72h**	0.3570	0.5495	
**100 µM L-AA 72h**	0.9786		
**5-(hydroxymethyl)-2'-deoxyuridine**			
	**1mM L-AA 72h**	**100 µM L-AA 72h**	**10 µM L-AA 72h**
**Control 72h**	**0.0002**	0.8020	1.0000
**10 µM L-AA 72h**	**0.0002**	0.7871	
**100 µM L-AA 72h**	**0.0002**		
**2'-deoxyuridine**			
	**1mM L-AA 72h**	**100 µM L-AA 72h**	**10 µM L-AA 72h**
**Control 72h**	0.6023	1.0000	0.6444
**10 µM L-AA 72h**	0.9998	0.6254	
**100 µM L-AA 72h**	0.5835		
**8-oxo-7,8-dihydro-2'-deoxyguanosine**			
	**1mM L-AA 72h**	**100 µM L-AA 72h**	**10 µM L-AA 72h**
**Control 72h**	**0.0116**	0.3080	0.2830
**10 µM L-AA 72h**	0.0959	**0.0473**	
**100 µM L-AA 72h**	**0.0014**		

Supplementary Table S8 Results of post-hoc test for comparisons of levels of DNA modifications in DNA of S2 cells after 72-hour cultivation in the presence of different concentrations of L-ascorbic acid.

	**modifications per 10^6 dN**		
	**5-(hydroxymethyl)-2'-deoxyuridine**	**2'-deoxyuridine**	**8-oxo-7,8-dihydro-2'-deoxyguanosine**
	mean±SD	mean±SD	mean±SD
	median (interquartile range)	median (interquartile range)	median (interquartile range)
**control 72h**	0.565±0.034	9.578±0.509	0.594±0.018
	0.550 (0.541;0.604)	9.659 (9.033;10.041)	0.586 (0.581;0.614)
**1 mM L-AA 24h**	2.792±0.153	10.733±2.394	1.049±0.090
	2.757 (2.661;2.960)	9.926 (8.846;13.425)	1.070 (0.950;1.126)
**1 mM L-AA 48h**	4.128±0.308	10.082±0.574	1.028±0.009
	4.204 (3.790;4.391)	10.114 (9.493;10.640)	1.029 (1.019;1.037)
**1 mM L-AA 72h**	5.187±0.921	9.645±0.764	1.116±0.200
	5.572 (4.136;5.852)	10.047 (8.764;10.125)	1.003 (0.999;1.347)

Supplementary Table S9 The levels of potential epigenetic DNA modifications in S2 cells cultured with addition of 1 mM L-AA for 24h, 48h and 72h.

5-(hydroxymethyl)-2’-deoxyuridine			
	**1mM L-AA 72h**	**1mM L-AA 48h**	**1mM L-AA 24h**
**control**	**<0.0001**	**<0.0001**	**0.0005**
**1 mM L-AA 24h**	**0.0003**	**0.0104**	
**1 mM L-AA 48h**	**0.0298**		
**2’-deoxyuridine**			
	**1mM L-AA 72h**	**1mM L-AA 48h**	**1mM L-AA 24h**
**control**	0.9999	0.9636	0.7125
**1 mM L-AA 24h**	0.7466	0.9273	
**1 mM L-AA 48h**	0.9757		
**8-oxo-7,8-dihydro-2’-deoxyguanosine**			
	**1mM L-AA 72h**	**1mM L-AA 48h**	**1mM L-AA 24h**
**control**	**0.0019**	**0.0057**	**0.0044**
**1 mM L-AA 24h**	0.8722	0.9957	
**1 mM L-AA 48h**	0.7643		

Supplementary Table S10 Results of post-hoc test for comparisons of levels of DNA modifications in DNA of S2 cells after culture with 1 mM L-ascorbic acid for 24h, 48h and 72h.

	modifications per 10^6 dN		
	**5-(hydroxymethyl)-2’-deoxyuridine**	**2’-deoxyuridine**	**8-oxo-7,8-dihydro-2’-deoxyguanosine**
	mean±SD	mean±SD	mean±SD
	median (interquartile range)	median (interquartile range)	median (interquartile range)
**control 24h**	2.364±0.382	8.226±0.846	2.061±0.628
	2.279 (2.102;2.386)	7.939 (7.861;8.263	1.946 (1.589;2.255)
**control 48h**	2.192±0.523	8.313±1.245	1.862±0.765
	2.113 (1.851;2.242)	8.057 (7.261;9.018)	1.554 (1.522;1.597)
**control 72h**	2.578±0.675	9.051±1.434	1.686±0.223
	2.355 (2.105;2.830)	8.436 (8.395;9.991)	1.667 (1.486;1.854)
**control 96h**	2.353±0.288	9.191±1.949	1.621±0.128
	2.324 (2.101;2.558)	8.975 (7.518;10.158)	1.592 (1.544;1.618)
**control 120h**	2.279±0.389	9.828±1.517	1.678±0.123
	2.327 (2.126;2.603)	9.431 (8.532;10.647)	1.722 (1.547;1.752)
**control 192h**	2.378±0.312	9.855±1.827	1.508±0.172
	2.323 (2.118;2.444)	9.907 (8.146;11.217)	1.510 (1.362;1.597)
**1 mM L-AA 24h**	4.188±1.255	11.190±3.602	2.183±0.153
	3.874 (3.248;4.357)	10.127 (8.421;13.646)	2.162 (2.055;2.312)
**1 mM L-AA 48h**	4.338±0.613	10.382±3.018	1.924±0.482
	4.307 (3.897;4.973)	9.522 (7.769;13.216)	1.805 (1.544;2.487)
**1 mM L-AA 72h**	6.125±1.169	15.373±7.825	2.009±0.379
	6.083 (5.388;6.448)	14.704 (8.393;21.812)	2.024 (1.648;2.385)
**1 mM L-AA 96h**	8.301±1.880	15.483±7.686	2.154±0.723
	8.316 (6.508;9.793)	14.534 (8.581;22.508)	2.027 (1.483;2.576
**1 mM L-AA 120h**	8.225±1.628	16.767±8.721	1.784±0.222
	8.037 (6.708;8.793)	15.881 (8.814;25.171)	1.776 (1.644;1.979)
**72h wash-out**	3.930±0.868	11.716±3.495	2.246±0.223
	3.909 (3.353;4.101)	9.541 (9.017;15.535)	2.296 (2.195;2.390)

Supplementary Table S11 Kinetics of DNA modification in S2 cells supplemented with 1 mM L-ascorbic acid and in control cells.

**5-hmdU**	**72h wash-out**	**1 mM L-AA 120h**	**1 mM L-AA 96h**	**1 mM L-AA 72h**	**1 mM L-AA 48h**	**1 mM L-AA 24h**	**Control 192h**	**Control 120h**	**Control 96h**	**Control 72h**	**Control 48h**
**Control 24h**	**0.0116**	**<0.0001**	**<0.0001**	**<0.0001**	**0.0011**	**0.0023**	0.9805	0.8819	0.9847	0.6206	0.7755
**Control 48h**	**0.0075**	**<0.0001**	**<0.0001**	**<0.0001**	**0.0007**	**0.0016**	0.7577	0.8858	0.7895	0.4558	
**Control 72h**	**0.0480**	**<0.0001**	**<0.0001**	**<0.0001**	**0.0072**	**0.0139**	0.6371	0.5247	0.6078		
**Control 96h**	**0.0111**	**<0.0001**	**<0.0001**	**<0.0001**	**0.0010**	**0.0022**	0.9652	0.8970			
**Control 120h**	**0.0080**	**<0.0001**	**<0.0001**	**<0.0001**	**0.0007**	**0.0015**	0.8626				
**Control 192h**	**0.0123**	**<0.0001**	**<0.0001**	**<0.0001**	**0.001151**	**0.0025**					
**1 mM L-AA 24h**	0.6696	**<0.0001**	**<0.0001**	**0.0013**	0.793606						
**1 mM L-AA 48h**	0.4996	**<0.0001**	**<0.0001**	**0.0028**							
**1 mM L-AA 72h**	**0.0006**	**0.0005**	**0.0004**								
**1 mM L-AA 96h**	**<0.0001**	0.8946									
**1 mM L-AA 120h**	**<0.0001**										

Supplementary Table S12 Results of post-hoc test for comparisons of level of 5-(hydroxymethyl)-2’-deoxyuridine in DNA of S2 cells supplemented with 1 mM L-ascorbic acid.

dU	72h wash-out	1 mM L-AA 120h	1 mM L-AA 96h	1 mM L-AA 72h	1 mM L-AA 48h	1 mM L-AA 24h	Control 192h	Control 120h	Control 96h	Control 72h	Control 48h
**Control 24h**	0.2790	0.5320	0.9177	0.9979	1.0000	0.9806	0.9982	0.6476	1.0000	1.0000	0.9991
**Control 48h**	0.0546	0.1387	0.4524	0.8103	0.9998	0.6377	0.8180	0.1949	1.0000	0.9766	
**Control 72h**	0.5011	0.7802	0.9905	1.0000	1.0000	0.9993	1.0000	0.8694	0.9974		
**Control 96h**	0.1029	0.2407	0.6421	0.9323	1.0000	0.8143	0.9365	0.3243			
**Control 120h**	0.9999	1.0000	1.0000	0.9894	0.5521	0.9992	0.9882				
**Control 192h**	0.8059	0.9653	1.0000	1.0000	0.9935	1.0000					
**1 mM L-AA 24h**	0.9320	0.9955	1.0000	1.0000	0.9563						
**1 mM L-AA 48h**	0.2161	0.4396	0.8601	0.9927							
**1 mM L-AA 72h**	0.8137	0.9680	1.0000								
**1 mM L-AA 96h**	0.9856	0.9998									
**1 mM L-AA 120h**	1.0000										

Supplementary Table S13 Results of post-hoc test for comparisons of level of 2’-deoxyuridine in DNA of S2 cells supplemented with 1 mM L-ascorbic acid.

8-oxodG	72h wash-out	1 mM L-AA 120h	1 mM L-AA 96h	1 mM L-AA 72h	1 mM L-AA 48h	1 mM L-AA 24h	Control 192h	Control 120h	Control 96h	Control 72h	Control 48h
**Control 24h**	0.9998	0.9907	1.0000	1.0000	1.0000	1.0000	0.4907	0.9047	0.7960	0.9633	0.9997
**Control 48h**	0.9462	1.0000	0.9901	1.0000	1.0000	0.9792	0.9590	0.9999	0.9982	1.0000	
**Control 72h**	0.6794	1.0000	0.8389	0.9891	0.9995	0.7770	0.9996	1.0000	1.0000		
**Control 96h**	0.3770	0.9999	0.5466	0.8972	0.9810	0.4650	1.0000	1.0000			
**Control 120h**	0.5229	1.0000	0.7048	0.9633	0.9965	0.6247	0.9999				
**Control 192h**	0.1615	0.9910	0.2611	0.6372	0.8478	0.2060					
**1 mM L-AA 24h**	1.0000	0.8791	1.0000	0.9999	0.9946						
**1 mM L-AA 48h**	0.9794	1.0000	0.9981	1.0000							
**1 mM L-AA 72h**	0.9984	0.9984	1.0000								
**1 mM L-AA 96h**	1.0000	0.9234									
**1 mM L-AA 120h**	0.7961										

Supplementary Table S14 Results of post-hoc test for comparisons of level of 8-oxo-7,8-dihydro-2’-deoxyguanosine in DNA of S2 cells supplemented with 1 mM L-ascorbic acid.

Supplementary Figure S2 Correlation between the level of 8-oxo-7,8-dihydro-2'-deoxyguanosine and 5-(hydroxymethyl)-2'-deoxyuridine.

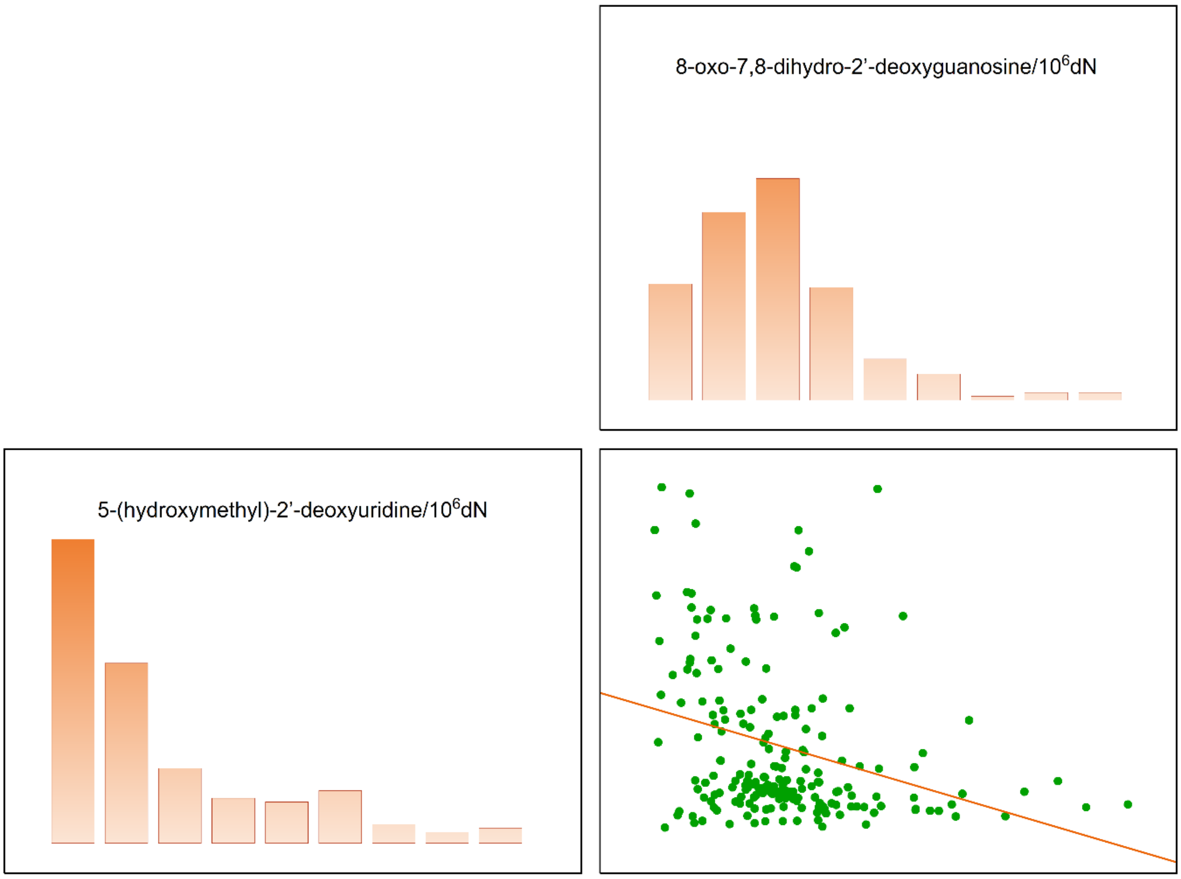

	**8-oxo-7,8-dihydro-2'-deoxyguanosine**
**5-(hydroxymethyl)-2'-deoxyuridine**	-0.3018
	N=193
	$p<0.0001$

For 100 mL of *Drosophila* medium		
Agar	4.5 g	Dissolved in water to final volume 100 mL
Yeast	1.5 g	
Sucrose	2.5 g	
Antibiotics	tetracycline 1.5 mg ampicillin 5 mg	Fresh solution of antibiotics
Tegosept	680 µL	10% p-hydroxy-benzoic acid methyl ester in 95% ethanol
Acid mix	1150 µL	For 50 mL of acid mix: 27.03 mL H_2O, 20.9 mL propionic acid, 2.07 mL phosphoric acid

Supplementary Table S15 *Drosophila* medium recipe.

	**Final concentrations of L-AA in medium**	**Incubation time**	**Comments**
Experiment 1	10 µM, 100 µM, 1 mM	72 h	medium supplemented with L-AA every 24 hours
Experiment 2	1 mM	24 h, 48 h, 72 h	medium supplemented with L-AA every 24 hours
Experiment 3	1 mM	24 h, 48 h, 72 h, 96 h, 120 h, 72h wash-out	medium supplemented with L-AA every 24 hours wash-out → cells were supplemented with L-AA for 120 h, then the medium was changed and cells were cultured for 72 h in non-supplemented medium

Supplementary Table S16 Description of experiments on *Drosophila* S2 cells.

Supplementary Methods

DNA extraction from Schneider 2 cells

DNA extraction from cells was performed according to a previously published protocol (Starczak et al.[1]). In brief, a pellet of frozen cells was dispersed in the ice-cold buffer B (Tris-HCl (10 mmol/L), Na_2EDTA (5 mmol/L) and deferoxamine mesylate (0.15 mmol/L), pH 8.0). Next 60 µL of RNase A (2 mg/mL, Sigma), 20 µL of RNase T1 (2 U/µL, Sigma) and SDS solution (to a final concentration of 0.5 %) were added, and the mixture was gently mixed using a polypropylene Pasteur pipette. The samples were incubated at 37 °C for 30 minutes. Proteinase K was added to a final concentration of 4 mg/mL and incubated at 37 °C for 1.5 h. The mixture was cooled, transferred to a centrifuge tube with phenol:chloroform:isoamyl alcohol (25:24:1), vortexed vigorously and centrifuged for 15 min at 2800 × g at 4 °C. After the extraction, the aqueous phase was treated with a chloroform:isoamyl alcohol mixture (24:1) and centrifuged under the same conditions. The supernatant was treated with three volumes of cold 96 % (v/v) ethanol to precipitate high molecular weight nucleic acids. The precipitate was removed with a plastic spatula, washed with 70 % (v/v) ethanol and dissolved in 50 µL of the Milli-Q grade deionized water.

Mass-spectrometry profiling of modified nucleotides

The analyses were performed using a method described earlier by Gackowski et al. [2] with some modifications. Briefly speaking, the chromatographic separation was performed with a Waters Acquity 2D-UPLC system with photo-diode array detector for the first dimension chromatography (used for the quantification of unmodified deoxynucleosides) and Xevo TQ-XS tandem quadrupole mass spectrometer (used for the second dimension chromatography and compounds analyzed after the first dimension: 5-hmdC, 5-mdC, 5-formyl-2'-deoxycytidine and 8-oxodG, to assure the better ionization at the higher acetic acid concentration). At-column-dilution technique was used between the first and second dimension to improve the retention at the trap/transfer column. The columns used were as follows: a Waters Cortecs T3 column (150 mm×3 mm, 1.6 µm) with a precolumn at the first dimension, a Waters X-select C18 CSH (100 mm×2.1 mm, 1.7 µm) at the second dimension and Waters X-select C18 CSH (20 mm×3 mm, 3.5 µm) as a trap/transfer column. The chromatographic system operated in a heart-cutting mode, indicating that selected parts of effluent from the first dimension were directed to the trap/transfer column via 6-port valve switching, which served as an "injector" for the second dimension chromatography system. The flow rate at the first dimension was 0.5 mL/min and the injection volume was 2 µL. The separation was performed with a gradient elution for 10 minutes using a mobile phase 0.05 % acetate (A) and acetonitrile (B) (0.7-5 % B for 5 minutes, followed by the column washing with 30 % acetonitrile and re-equilibration with 99 % A for 3.6 minutes). The flow rate at the second dimension was 0.35 mL/min. The separation was performed with a gradient elution for 10 minutes using a mobile phase 0.01 % acetate (A) and methanol (B) (1-50 % B for 4 minutes, isocratic flow of 50 % B for 1.5 minutes, and re-equilibration with 99 % A up to the next injection). All the samples were analyzed in three to five technical replicates the technical mean of which was used for further calculation. Mass spectrometric detection was performed using the Waters Xevo TQ-XS tandem quadrupole mass spectrometer, equipped with an electrospray ionization source. Collision-induced dissociation was obtained using argon 6.0 at 3 x 10-6 bar pressure as the collision gas. Transition patterns for all the analyzed compounds along with specific detector settings were determined using the MassLynx 4.2 Intelli-Start feature in a quantitative mode to assure the best signal-to noise ratio and the resolution of 1 at MS1 and 0.75 at MS2 (Supplementary Table S17).

compound name		ionization mode	nominal molecular mass (Da)	pseudomolecular ion formulation	nominal parent ion (Da)	nominal daughter ion (Da)	ESI capillary (kV)	ESI cone (V)	collision energy (eV)	standard source
5-(hydroxymethyl)-2'-deoxycytidine	quantifier	positive	257	$[M+H]^+$	258	124	1.2	15	10	Berry & Associates
[D_3]-5-(hydroxymethyl)-2'-deoxycytidine	quantifier	positive	260	[(M+3)+H]+	261	127	1.2	15	10	Toronto Research Chemicals
5-formyl-2'-deoxycytidine	quantifier	negative	255	[M-H]-	254	121	3.5	28	18	Berry & Associates
[$^{13}C_{10}$, $^{15}N_2$]-5-formyl-2'-deoxycytidine	quantifier	negative	267	[(M+12)-H]-	266	128	3.5	28	18	own synthesis
5-carboxy-2'-deoxycytidine	quantifier	negative	271	[M-H]-	270	110	3.5	20	20	Berry & Associates
[$^{13}C_{10}$, $^{15}N_2$]-5-carboxy-2'-deoxycytidine	quantifier	negative	283	[(M+12)-H]-	282	116	3.5	20	20	own synthesis
5-(hydroxymethyl)-2'-deoxyuridine	quantifier	negative	258	[M-H]-	257	124	3.5	20	15	Berry & Associates
[$^{13}C_{10}$, $^{15}N_2$]-5-(hydroxymethyl)-2'-deoxyuridine	quantifier	negative	270	[(M+12)-H]-	269	131	3.5	20	15	own synthesis
2'-deoxyuridine	quantifier	negative	228	[M-H]-	227	184	3.5	20	12	Sigma-Aldrich
[^{13}C, $^{15}N_2$]-2'-deoxyuridine	quantifier	negative	231	[(M+3)-H]-	230	185	3.5	20	12	Medical Isotopes
8-oxo-7,8-dihydro-2'-deoxyguanosine	quantifier	negative	283	[M-H]-	282	192	1.2	20	15	Sigma-Aldrich
[$^{15}N_5$]-8-oxo-7,8-dihydro-2'-deoxyguanosine	quantifier	negative	288	[(M+5)-H]-	287	197	1.2	20	15	Cambridge Isotope Laboratories
5-methyl-2'-deoxycytidine	quantifier	positive	241	[M+H]+	242	126	1.2	12	18	Jena Bioscience
[$^{13}C_{10}$, $^{15}N_2$]-5-methyl-2'-deoxycytidine	quantifier	positive	253	[(M+12)+H]+	254	133	1.2	12	18	own synthesis
N6-methyl-2’-deoxyadenosine	quantifier	positive	265	[M+H]+	266	150	3.5	15	15	Toronto Research Chemicals
[D_3]-N6-methyl-2’-deoxyadenosine	quantifier	positive	268	[(M+3)+H]+	269	153	3.5	15	18	Toronto Research Chemicals

Supplementary Table S17 Transition patterns-specific detector settings and sources of standards for the analysed compounds.

Supplementary Methods

Analysis of L-ascorbic acid levels in cells and culture medium

In order to analyze the concentration of L-AA, both in the culture medium and inside the cells, the previously described ultra-performance liquid chromatography (UPLC) with the UV detection method was used (Modrzejewska et al. [3]). In brief cells were suspended in 25 µL of cold phosphate-buffered saline (PBS), and 25 µL of precooled 10% (w/v) meta-phosphoric acid aqueous solution were added to obtain L-AA stabilization and the cell membrane perforation. Also 50 µL aliquots of medium samples were treated in the same manner. Then, cells suspension was sonicated for 5 min and incubated on ice for 30 min. Next, the samples were diluted 1:1 with Milli-Q grade deionized water, vortexed and centrifuged at 24 400 × g for 15 min at 4 °C. The supernatants were purified by ultrafiltration using AcroPrep Advance 96-Well Filter Plates 10 K and injected into Waters Acquity UPLC system. The method was validated using the reference material from Chromsystems. The intracellular concentration of ascorbate was calculated under the assumption that mean diameter of S2 cell equals to 9.9±0.33 µm.

The samples were analyzed on Waters Acquity UPLC HSS T3 column (150 mm×2.1 mm, 1.8 µm) with a flow rate 0.25 mL/min and 2 µL injection volume. Ammonium formate (10 mM, pH 3.1) was used as Solvent A and acetonitrile was Solvent B. The following program was used for the ascorbate elution: 0–0.1 min 99 % A, 1 % B, 0.1–2.2 min 97 % A, 2.2–4.0 min – linear gradient to 90 % A, 4.0–4.5 min – 90 % A, 4.5–6.0 min – 99 % A. The column thermostat was set at 10 °C. The effluent was monitored with a photo-diode array detector at 254 nm, and analyzed with Empower software.

Time (min)	Flow (ml/min)	%A	%B	Curve
Initial	0.300	99.9	0.1	Initial
0.20	0.300	99.9	0.1	6
1.20	0.300	80.0	20.0	6
1.50	0.300	70.0	30.0	6
2.50	0.300	70.0	30.0	6
2.60	0.300	99.9	0.1	6

Supplementary Table S18 Flow gradient used for elution during determination of intracellular L-AA concentration in *D. melanogaster.*

Supplementary Methods

Determination of thymine in acidic hydrolysates of *Drosophila* extracts

The determination of thymine in homogenates from three developmental stages of *D. melanogaster* (larvae, pupae, imago) was performed using the method described by Modrzejewska et al.[4] with some modifications. Namely, 20 µL of insect homogenate was incubated at 130 °C for 1 h with 200 µL of 515 µM caffeine in 2 M HCl in a sealed 2 mL glass vial. The cooled sample was completely dried under nitrogen (XcelVap, Biotage AB), dissolved in 50 µL of the Milli-Q grade deionized water and ultrafiltered prior to the injection. A 2 µL aliquot of the sample was chromatographed in duplicate at a flow rate of 0.45 mL/min and 30 °C on CORTECS UPLC T3 1.6 µm (3 x 150 mm) column coupled to Waters Acquity UPLC system with a photo-diode array detector, using two solvents: A - 10 mM ammonium formate (pH 3.14) and B - acetonitrile, according to the following elution program: 0–0.1 min, isocratic, 0.1 % B; 0.1–2 min, linear gradient 0.1 %–15 % B; 2-3 min min, linear gradient 15 %–50 % B; 3–3.5 min, isocratic, 50 % B; 3.5–3.51 min, linear gradient, 50 %–0.1 % B. The effluent was monitored with a photo-diode array detector at 254 nm, and analyzed with the Empower software.

Supplementary References

1 Starczak, M., Gawronski, M., Olinski, R. & Gackowski, D. Quantification of DNA Modifications Using Two-Dimensional Ultraperformance Liquid Chromatography Tandem Mass Spectrometry (2D-UPLC-MS/MS). *Methods Mol Biol* **2198**, 91-108, doi:10.1007/978-1-0716-0876-0_8 (2021).

2 Gackowski, D. *et al.* Accurate, Direct, and High-Throughput Analyses of a Broad Spectrum of Endogenously Generated DNA Base Modifications with Isotope-Dilution Two-Dimensional Ultraperformance Liquid Chromatography with Tandem Mass Spectrometry: Possible Clinical Implication. *Anal Chem* **88**, 12128-12136, doi:10.1021/acs.analchem.6b02900 (2016).

3 Modrzejewska, M. *et al.* Vitamin C enhances substantially formation of 5-hydroxymethyluracil in cellular DNA. *Free Radical Bio Med* **101**, 378-383, doi:10.1016/j.freeradbiomed.2016.10.535 (2016).

4 Modrzejewska, M., Gawronski, M. & Gackowski, D. Normalization of metabolic data to total thymine content and its application to determination of 2-hydroxyglutarate. *Anal Biochem* **618**, 114129, doi:10.1016/j.ab.2021.114129 (2021).
